# Structure of ubiquitylated-Rpn10 provides insight into its autoregulation mechanism

**DOI:** 10.1038/ncomms12960

**Published:** 2016-10-04

**Authors:** Tal Keren-Kaplan, Lee Zeev Peters, Olga Levin-Kravets, Ilan Attali, Oded Kleifeld, Noa Shohat, Shay Artzi, Ori Zucker, Inbar Pilzer, Noa Reis, Michael H. Glickman, Shay Ben-Aroya, Gali Prag

**Affiliations:** 1 Department of Biochemistry and Molecular Biology and the Institute of Structural Biology, George S. Wise Faculty of Life Sciences, Tel Aviv University, Tel Aviv 69978, Israel; 2 Faculty of Life Sciences, Bar-Ilan University, Ramat-Gan 52900, Israel; 3 Department of Biochemistry and Molecular Biology, Monash University, Clayton, Victoria 3800, Australia; 4 Department of Biology, Technion—Israel Institute of Technology, Haifa 32000, Israel; 5 Sagol School of Neuroscience, Tel Aviv University, Tel Aviv 69978, Israel

## Abstract

Ubiquitin receptors decode ubiquitin signals into many cellular responses. Ubiquitin receptors also undergo coupled monoubiquitylation, and rapid deubiquitylation has hampered the characterization of the ubiquitylated state. Using bacteria that express a ubiquitylation apparatus, we purified and determined the crystal structure of the proteasomal ubiquitin-receptor Rpn10 in its ubiquitylated state. The structure shows a novel ubiquitin-binding patch that directs K84 ubiquitylation. Superimposition of ubiquitylated-Rpn10 onto electron-microscopy models of proteasomes indicates that the Rpn10-conjugated ubiquitin clashes with Rpn9, suggesting that ubiquitylation might be involved in releasing Rpn10 from the proteasome. Indeed, ubiquitylation on immobilized proteasomes dissociates the modified Rpn10 from the complex, while unmodified Rpn10 mainly remains associated. *In vivo* experiments indicate that contrary to wild type, Rpn10-K84R is stably associated with the proteasomal subunit Rpn9. Similarly Rpn10, but not ubiquitylated-Rpn10, binds Rpn9 *in vitro*. Thus we suggest that ubiquitylation functions to dissociate modified ubiquitin receptors from their targets, a function that promotes cyclic activity of ubiquitin receptors.

## Abstract (web summary)

Ubiquitin (Ub) receptors are responsible for the recognition of ubiquitylated proteins. Here the authors describe the crystal structure of the ubiquitylated form of the Ub-receptor Rpn10, which suggest that ubiquitylation of Rpn10 promotes its dissociation from the proteasome.

## Graphical abstract

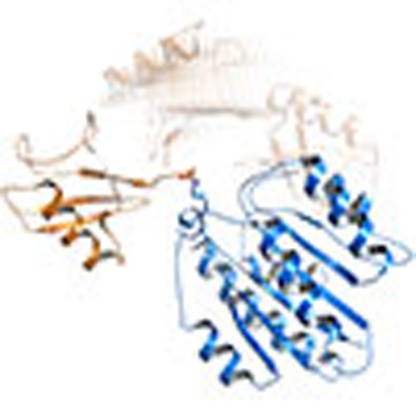

Hundreds of ubiquitin (Ub)-receptors recognize and decode diverse Ub-signals into cellular responses^1^,^2^. Typically, Ub-receptors contain three modules: a Ub-binding module(s) that recognize the Ub-signal, a response module that decodes the Ub-signal into a specific cellular response and a ‘context' module that spatially and timely targets the Ub-receptor to its site of action. Ub-receptors themselves can undergo auto-monoubiquitylation^3^,^4^. However, the function of this self-ubiquitylation remains unknown. One possibility is that ubiquitylation inhibits Ub-receptor activity by blocking its Ub-binding domain (UBD)^1^,^5^,^6^,^7^. Determination of the structure of the ubiquitylated state is expected to elucidate the significance of Ub-receptor ubiquitylation. Until recently, however, the ability to purify proteins in a homogenous ubiquitylated state was limited due to their rapid deubiquitylation. Indeed, among nearly a thousand reported structures of Ub-receptors, none are of the ubiquitylated state.

Here we circumvent this limitation by purifying large quantities of genuine ubiquitylated substrates from deubiquitylases-free bacteria exogenously expressing the ubiquitylation apparatus^5^, applying it to the proteasomal Ub-receptor Rpn10 as a model. Rpn10 decodes K48-linked poly-Ub-signals into proteasomal degradation^8^,^9^,^10^. It was previously demonstrated that Rpn10 undergoes Rsp5-dependent monoubiquitylation on the proteasome, followed by deubiquitylation^7^. Rpn10 is loosely associated with proteasomes, but the structural mechanism that releases this Ub-receptor from the proteasome was never elucidated. Large pools of a proteasome-free Rpn10 have been observed in the cytosols of several model organisms^10^,^11^,^12^, and has been postulated that these shuttle ubiquitylated substrates or other Ub-shuttles, including Rad23, Dsk2 and Ddi1, to the proteasome^11^,^13^. Indeed, ectopic expression of Rpn10-ubiquitin interacting motifs (UIMs) resulted in accumulation of cytosolic ubiquitylated substrates, supporting the shuttling model^14^,^15^.

Here we report the structure of ubiquitylated-Rpn10 (Ub-Rpn10). The structure reveals novel UBD features and suggests a mechanism for dissociation of Ub-Rpn10 from proteasomes. Together with *in vivo* and *in vitro* data, we propose that the ubiquitylation of Ub-receptors functions to dissociate them from their target to promote cyclic activity.

## Results

### Structure of Ub-Rpn10

**Figure 1:**
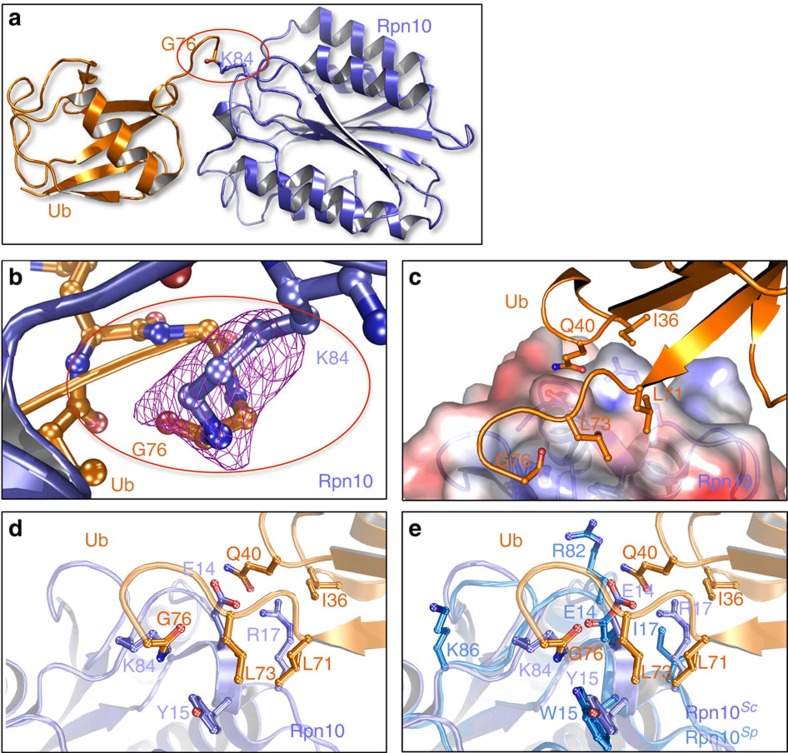
Structure of ubiquitylated-Rpn10. (**a**) Structure of Ub-Rpn10. Ub (orange) and Rpn10 (purple). (**b**) The refined model of the isopeptide linkage between Rpn10-K84 and Ub-G76 is shown with a Sigma-A m*Fo-DFc* simulated-annealing omit map contoured at 2.0 *σ* units. (**c**) Electrostatic surface representation of the ubiquitylation site vicinity. The surface is coloured according to the electrostatic potential (±15 kT) and calculated in the absence of Ub with the program ABPS. (**d**) Ball-and-stick model showing the interactions between Ub and the vWA domain at the ubiquitylation site. The orientation is the same as in **c**. (**e**) Structural alignment of the interaction area surrounding K84 in Rpn10 from *S. cerevisiae* (purple) and from *S. pombe* (blue) shows that the two proteins share similar binding characteristics (supplementary Fig. 1).

We purified monoubiquitylated-Rpn10 from bacteria that express functional ubiquitylation apparatus using our previously described system^5^. The protein was crystalized^16^ and the structure determined to a resolution of 3.1 Å (Fig. 1a; Table 1). Out of hundreds of structures of Ub-receptors and their non-covalent complexes with Ub, this is the first structural report of a ubiquitylated-Ub-receptor. The ubiquitylation site was clearly observed at K84, a residue previously identified *in vivo* as the major ubiquitylation site^7^. Omit map analysis revealed an *mFo-DFc* electron density >3.0 *σ* around the isopeptide bond^17^ (Fig. 1b). The structure shows several non-covalent interactions near the isopeptide bond, which are mediated via a region in the vicinity of Ub-I36, a well-known patch previously shown to interact with other UBDs^18^. This interaction buries a surface of 418 Å^2^, a typical value found in other UBDs^1^. This attraction stabilizes the usually flexible C terminus tail of Ub, which assumes a shrunk conformation. Two clefts located in close proximity to K84 form the interface with Ub (Fig. 1c). Rpn10 residues E14 and R17, located on the ridge between these clefts, electrostatically interact with Ub-Q40. The aliphatic arms of residues E14 and R17, together with Y15, face the hydrophobic patch on Ub that includes residues I36, L71 and L73 (ref. 18; Fig. 1d).

Superposition of *Saccharomyces cerevisiae* Ub-Rpn10 on the structure of *Schizosaccharomyces pombe* Rpn10 (ref. 19) yielded an r.m.s.d. value of 0.85 Å, suggesting that ubiquitylation did not induce significant conformational changes. The alignment shows that the two proteins present a highly conserved surface surrounding K84 (Fig. 1e; Supplementary Fig. 1). Interestingly, the structural comparison uncovered compensatory substitutions of R17/I17 and E82/R82 of the *S. cerevisiae*/*S. pombe* residues at the ubiquitylation site interface.

### The vWA domain is a bona fide UBD

**Figure 2:**
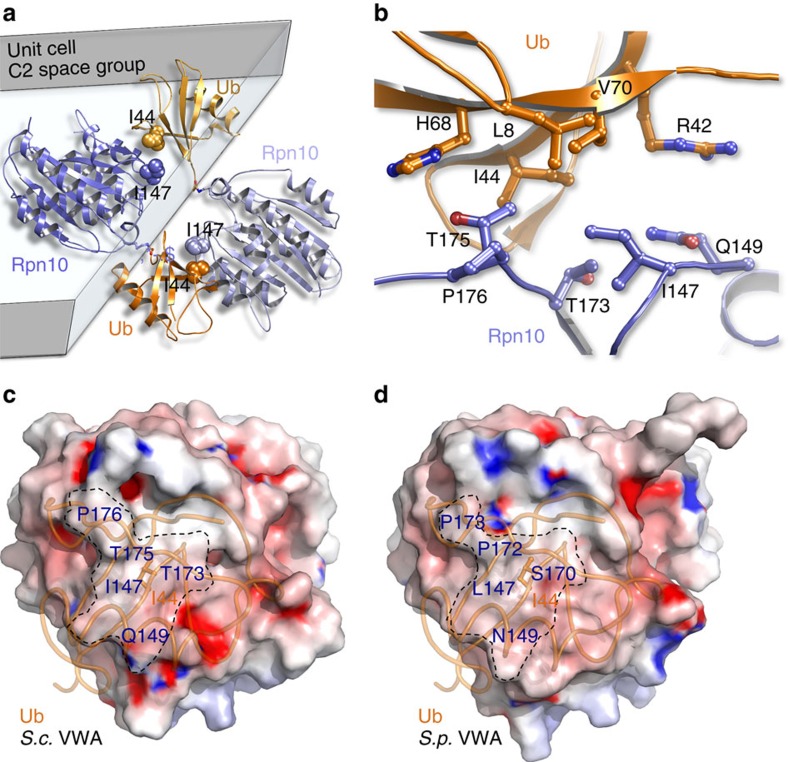
vWA and Ub form a non-covalent interface. (**a**) A non-covalent interface formed between the vWA domain (purple/light purple) and Ub (orange/light orange) in the crystal lattice. The residues Ub-I44 and Rpn10-I147 are presented as spheres. The unit cell faces are rendered in grey. (**b**) Detailed representation of the non-covalent-binding interface between Ub and Rpn10-vWA domain. (**c**,**d**) Electrostatic surface representation of the non-covalent Ub-binding patch of Rpn10 from *S. cerevisiae* (*Sc*; **c**) compared with *S. pombe* (*Sp*; **d**). The surface is coloured according to the electrostatic potential (±15 kT) and calculated in the absence of Ub with the program ABPS. The orientation of the Ub molecule with respect to the *S. pombe* vWA domain was achieved by superimposing the structures of these two proteins. Ub is rendered as an orange ribbon. Some of the interacting residues are indicated.

Rpn10 is composed from an N-terminal vWA (Von Willebrand factor type A) domain that was shown to participate in proteasome binding, and a C-terminal Ubiquitin Interacting Motif (UIM) domain that binds Ub through Ub-I44 hydrophobic patch. Inspection of the Ub-Rpn10 crystal lattice reveals non-covalent interactions between the vWA domain and the adjacent Ub-I44 hydrophobic patch from a symmetry related Ub-Rpn10 molecule (Fig. 2a). To ensure that we properly traced the C-termini of the covalently attached Ub molecule, we performed an unbiased refinement using simulated-annealing refinement of the partial structure^17^. The Ub C terminus segment (residues R72-G76) and the side-chain atoms of K84 (Cβ, Cγ, Cδ, Cɛ and Nζ) were omitted from the refinement process. A clear continuous electron density (of the 3.0 *σ*_*A*_-weighted *mFo-DFc* omit map) was found to cover the entire omitted segment, indicating we correctly traced the structure (Supplementary Fig. 2). The non-covalent interaction of the Ub-I44 patch with vWA suggests that the vWA domain may also function as a UBD^1^,^20^. We then employed our structural-based *in silico* algorithm to assess the biological significance of this proposed interaction^21^. Probing the entire surface of the vWA domain with physico-chemical properties of known UBDs (such as the UBA domain of E2-25k) suggests that Ub binds to a highly similar patch centered at I147 (Supplementary Figs 3 and 4).

Detailed examination of this postulated biological interface shows a network of hydrophobic interactions between I147 of Rpn10 and Ub-I44 patch (Fig. 2b). In addition, Rpn10 L172, P176, P178 and the methyl groups of T173 and T175 form hydrophobic interactions with Ub residues L8, I44, G47 and V70. Like most UBD:Ub complexes, the hydrophobic interface is surrounded by polar interactions. These include Rpn10 Q149 (which interacts with Ub R42 and Q49), and Rpn10 T173 and T175 (which form polar interactions with Ub G47 and H68, respectively). Together, these residues form a contact interface that buries an area of 398 Å^2^. Similarly, many other UBDs contain a very small Ub-binding surface, ranging in size from 389 Å^2^ in the case of the Npl4-NZF:Ub (1Q5W) to 923 Å^2^ in the case of the Vps9p-CUE:Ub (1P3Q) complexes^1^,^18^,^22^. The relatively small sizes of the binding interfaces are correlated with the low affinity they possess, the small molecular weight of Ub, and their transient Ub-binding function^23^.

The sequences of Rpn10-vWA domains of *S. cerevisiae, S. pombe* and humans are highly conserved (Supplementary Fig. 1). However, structures are available only for the *S. cerevisiae* and the *S. pombe* proteins^19^,^24^,^25^. A structural comparison between them shows that the predicted Ub-binding patch is highly conserved. Specifically, I147 in *S. cerevisiae* is conservatively replaced with leucine in *S. pombe*. P176 is conserved as P173, and Q149 is conservatively altered to asparagine. The physico-chemical aliphatic properties of the Cβ and Cγ atoms of T173 and T175 are structurally maintained by S170 and P172 of *S. pombe*, respectively. Surface representations of the electrostatic potential at the predicted Ub-binding patches show that the two proteins are highly similar in terms of the charges versus hydrophobic distribution (Fig. 2c,d). Together, these data suggest that the newly identified Ub-binding patch on vWA is evolutionarily conserved.

**Figure 3:**
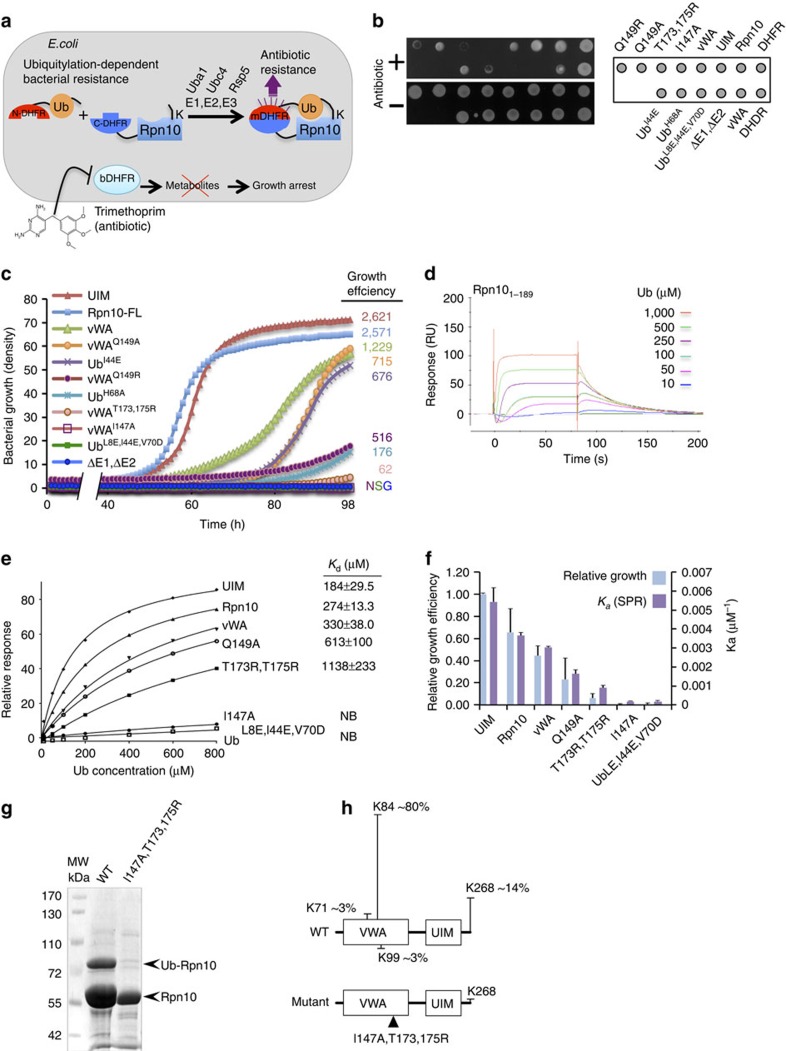
Functional analysis of the vWA:Ub-binding patch. (**a**) Scheme showing a bacterial genetic selection system for ubiquitylation. (**b**) Ubiquitylation-addicted bacterial growth on selective (+Trimethoprim) or non-selective plates. A single scan of the plates 98 h post seeding is shown (for time-lapse live-scan movies, see Supplementary Movies 1 and 2). (**c**) Quantification of ubiquitylation-dependent bacterial growth. Average density of individual spots monitored by scanning the plates in 1-h intervals was plotted. Efficiency was calculated as the maximum growth density divided by the time of half max growth. NSG, no significant growth. (**d**) A representative SPR response curves for the vWA:Ub complex. (**e**) Single model binding analysis of SPR affinity measurements of Rpn10:Ub wt and the indicated mutants. *K*_d_ values are indicated (right; NB, no binding). (**f**) Comparison between the relative growth of wt or mutant spots and the SPR measured association constants. Pearson product-moment correlation coefficient is equal to 0.955. (**g**) A representative SDS–PAGE comparing the ubiquitylation amounts in purified wild-type and GST-Rpn10 mutant at the non-covalent Ub-binding patch. (**h**) Mass spectrometry analysis of the ubiquitylation sites for full-length wild-type Rpn10 and a mutant at the vWA:Ub non-covalent-binding patch. The peaks were scaled according to the relative quantities of the detected GG peptides. For each ubiquitylation site, the total number of detected GG peptides and their percentage in the population are shown (Supplementary Figs 3 and 4).

To assess the structural-driven hypothesis that vWA functions as a UBD, we developed a genetic selection system for ubiquitylation in *Escherichia coli* lacking deubiquitylases (Fig. 3a). Fragments of a split antibiotic resistance gene (mouse DHFR (mDHFR)^26^) were tethered onto Ub and ubiquitylation substrate and were co-expressed with their cognate ubiquitylation apparatus (E1, yeast Ubc4 and Rsp5). Rpn10 ubiquitylation resulted in a stable covalent assembly of the antibiotic resistance protein, giving rise to bacterial growth on selective media (minimal media supplemented with the antibiotic Trimethoprim that selectively inhibits the bacterial DHFR (bDHFR)). We demonstrated the system functionality in identifying and characterizing new ubiquitylation components. As UBDs promote self-ubiquitylation^3^,^4^,^18^, the selection system provides a straightforward tool for their identification and characterization even at millimolar affinity. Indeed, bacteria co-expressing full-length Rpn10 or only its UIM and a functional ubiquitylation apparatus grew on selective and non-selective plates (Fig. 3b). Particularly, a construct containing only the vWA domain also presented a positive growth phenotype suggesting that vWA binds Ub. Structural-based mutational analyses of the vWA:Ub interface demonstrated the contribution of specific amino acids to the binding (Fig. 3b). We quantified the growth by measuring the bacterial density using time-lapse scanning and automated image analysis with Fiji (Fig. 3c; and a time-lapse movies 1–2 in the extended data). Strikingly, the bacterial genetic data is in excellent agreement with the structural information. We found that wild-type full-length Rpn10, UIM or vWA domains presented a higher productive growth phenotypes compared with the vWA:Ub interface point mutants. Moreover, like bacteria that expressed an incomplete ubiquitylation apparatus (ΔE1, ΔE2), the vWA-I147A or the UbL8E, I44E, V70D mutants presented highly significant growth arrest phenotypes.

To biophysically characterize the binding interface between the vWA domain and Ub, and to assess the structural model derived from the X-ray diffraction, we mutated the predicted vWA:Ub-binding interface. We then performed surface plasmon resonance (SPR) with the wild-type and mutant proteins to quantify their affinity to mono-Ub. Rpn10 derivatives (ligands) were immobilized, and increasing concentrations of free mono-Ub variants as analytes. The SPR analysis confirmed that the full-length Rpn10 or its UIM alone bind mono-Ub with affinities of ∼270 and ∼180 μM, respectively (Fig. 3d,e). Notably, the vWA domain alone bound mono-Ub with *K*_d_ of ∼330 μM, an affinity value that is in the range of many known UBDs^1^. The low affinities of UBDs, including the Rpn10 UIM and vWA, result in high noise of the measured SPR response units (RU). Moreover, the relatively small difference in the affinity of the UIM and the vWA, together with the high RU noise, preclude deconvolution of the binding curves. We therefore report a single-site-binding model for the full-length Rpn10, which presents a quasi-averaging of the affinities of the two Ub-binding sites. We further analysed the vWA:Ub-binding interface by measuring the affinity of the structure-based point mutants. The vWA-I147A mutant, that faces Ub-I44 (Fig. 2), completely abrogated binding. Mutating the surrounding residues T173R, T175R diminished binding affinity threefold, and mutating Q149, a residue predicted to have little contribution to the binding, resulted in a binding affinity 1.85-fold lower than that of the wild-type vWA. No binding response was measured for the Ub^L8E,I44E,V70D^ mutant, confirming that, as observed in the structure, vWA recognizes Ub similarly to most other UBDs. Size exclusion chromatography analysis showed that this Ub mutant was properly folded (Supplementary Fig. 5). We observed an excellent correlation between the growth efficiency under restrictive conditions of wild type and mutants in the genetic selection system and the binding affinities measured by the SPR. Figure 3f shows a comparison between the relative growth efficiency on selective media and the relative SPR association constants (*K*_a_). Linear regression provides a Pearson correlation coefficient of *r*=0.99 (*P*<0.001).

Taken together, these results demonstrate that vWA functions as a bona fide UBD and point to I147 as the centre of the binding patch.

### Non-covalent-binding targets ubiquitylation to K84

Rsp5 is the cognate E3 ligase of Rpn10 (ref. 27). Ubiquitylated Rsp5/NEDD4 protein family members interact with UIM-containing substrates to ubiquitylate them^3^. Therefore, it is predicted that removal of the UIM from Rpn10 will abolish its ubiquitylation. Indeed, in agreement with Crosas and co-workers^7^, we found a significantly decreased ubiquitylation of Rpn10 lucking its UIM (Fig. 3g). Similarly, we previously demonstrated that our bacterial ubiquitylation system preferentially selects K84 for ubiquitylation, as was previously found *in vivo* in yeast^5^,^7^. In light of this evidence, we hypothesized that one of the functions of the newly discovered Ub-binding patch on the vWA is to orient the Ub C terminus to target K84 for ubiquitylation. To test this hypothesis, we purified Rpn10 mutated at the Ub-binding patch from bacteria also expressing its cognate ubiquitylation apparatus (that is, Ub, Uba1, Ubc4, Rsp5 and Rpn10). Figure 3g presents a comparison of the ubiquitylation pattern of wild-type versus Rpn10 mutant at the hydrophobic I147 patch. It shows that the mutation resulted in a highly decreased degree of ubiquitylation, signifying the importance of the Ub-binding patch in the ubiquitylation process.

We then subjected this mutant protein to mass spectrometry (MS) analysis (Fig. 3h; Supplementary Figs 6 and 7). As expected, we found that K84 was the major ubiquitylation site of wild-type Rpn10, as previously reported^5^,^7^. Moreover, among the GG-K84 detected peptides of wild-type Rpn10, we also found a subpopulation of oxy-methionine peptides (at M87). This finding further indicates K84 as the main ubiquitylation site, as the methionine oxidation cross-validates with the ubiquitylation.

We did not detect ubiquitylation on K84 in the I417 Ub-binding patch mutant. Similarly, the residual ubiquitylation at K71 and K99 was not detected. However a few GG-isopeptides, corresponding only to ubiquitylation of K268 were detected. The significant reduction of the GG-isopeptides is in agreement with the Coomassie stained SDS–polyacrylamide gel electrophoresis analysis (Fig. 3g). Taken together, these data show that the newly identified Ub-binding patch on the vWA domain directs K84 ubiquitylation.

### Ub-Rpn10 in the context of the proteasome

**Figure 4:**
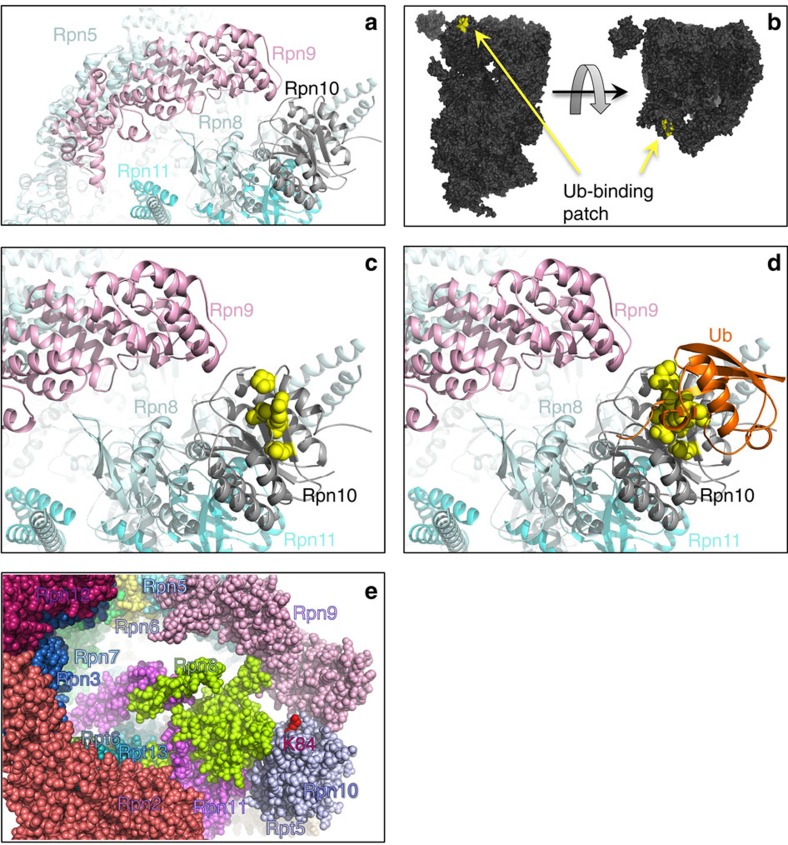
vWA Ub-binding patch and K84 are exposed in the proteasomal context. (**a**) Rpn10 loosely interacts with neighbouring proteasome subunits. (**b**) The vWA:Ub-binding patch is exposed in the proteasomal context. The proteasome surface is rendered in dark grey; the Ub-binding patch is in yellow. (**c**) Zoom-in into the non-covalent Ub-binding patch at the proteasome. The side chains of residues participating in Ub binding are rendered as spheres and coloured yellow. (**d**) Ub bound to the non-covalent Ub-binding patch based on the crystal lattice. Ub residues that participate in binding are also rendered as yellow spheres. (**e**) Revealing Rpn10-K84 as exposed in the context of the proteasome.

We next aimed to decipher the function of Rpn10 ubiquitylation based on our structure in the context of the proteasome. High-resolution EM structures of the proteasome show that the Rpn10-vWA domain weakly interacts with Rpn8 and Rpn9, and barely leans on Rpn11 (refs 25, 28, 29, 30; Fig. 4a). Calculations show that the surface area buried by Rpn9 is 2.5-fold larger than the area buried by Rpn10, (Supplementary Fig. 8). This supports the hypothesis that Rpn10 association with the proteasome is dynamic and reversible^8^,^10^,^11^. To obtain insight into the function of Rpn10 ubiquitylation in the proteasomal context, we superimposed the structures of the non-covalent vWA:Ub complex and of Ub-Rpn10 on several such cryo-EM structures of the entire proteasome^25^,^28^,^29^,^30^. Interestingly, we found that the Ub-binding patch on the vWA domain is completely exposed in the proteasome context (Fig. 4b). Such a structure may facilitate binding of ubiquitylated-cargo, as well as allow binding of ubiquitylated and catalytically loaded Rsp5 to direct Rpn10 ubiquitylation on K84 in the proteasome context. Indeed, it has been shown that isolated proteasome fractions contain a small population of monoubiquitylated-Rpn10, suggesting that Rpn10 ubiquitylation can occur on the proteasome^7^. This scenario would require both the Ub-binding patch and K84 to be exposed and accessible to Ub-loaded Rsp5 (Ub∼Rsp5, Fig. 4c). Superimposing the non-covalently interacting Ub from our structure (vWA:Ub complex) onto Rpn10 in the proteasome context reveals that Ub binding would not result in clashes with other proteasomal subunits (Fig. 4d). Therefore, this patch is in a position to recruit and orient Ub∼Rsp5 for K84 ubiquitylation.

Although K84 is also fully exposed, it is located at the edge of the Rpn10:Rpn9 interface and faces Rpn9 (Fig. 4e). The C terminus of the non-covalently bound Ub can therefore approach K84. Taken together, these findings support a hypothesis where ubiquitylation regulates Rpn10 function within the proteasome.

### Ub-Rpn10 clashes into Rpn9 in the context of the proteasome

**Figure 5:**
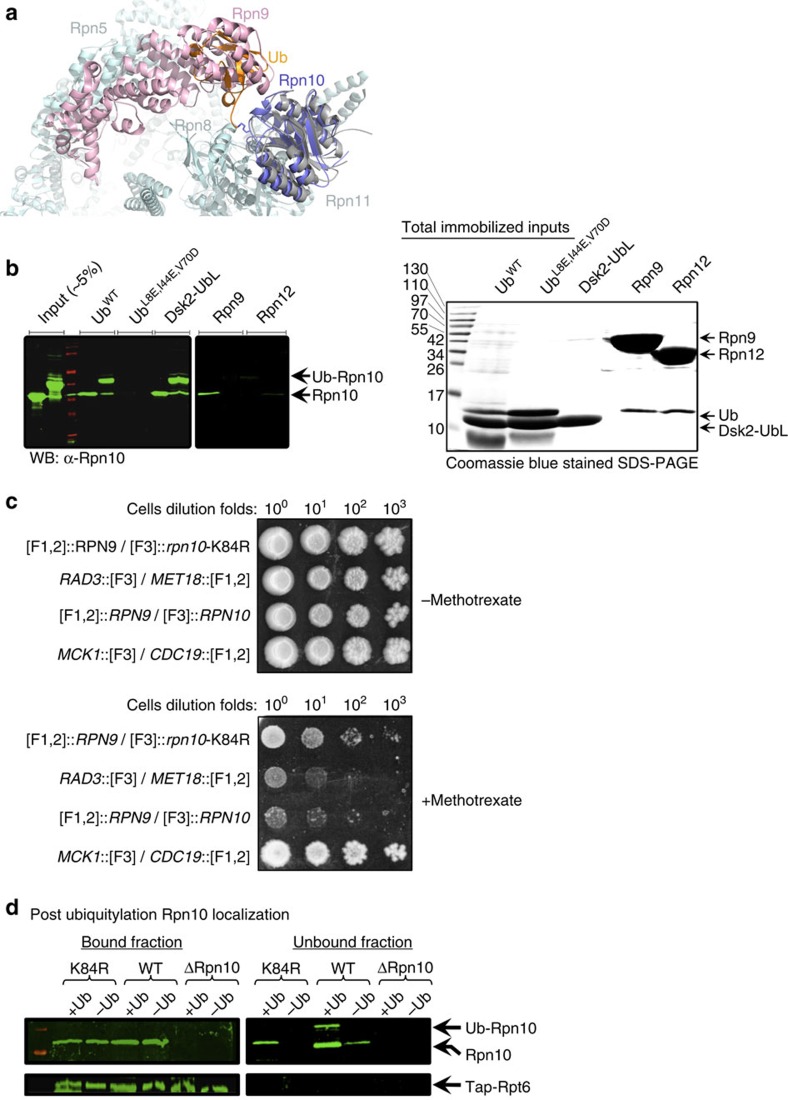
Ubiquitylation releases Rpn10 from the proteasome. (**a**) Superimposition of the Ub-Rpn10 onto the cryo-EM structure^23^. Colour code: grey, cryo-EM model; purple current model. The Ub moiety from the Ub-Rpn10 molecule (orange) clearly clashes into the Rpn9 subunit (pink). (**b**) Pull-down experiments of Rpn10 and Ub-Rpn10 by the indicated proteins. His_6_-tagged proteins were immobilized on Ni^2+^ beads and used to pull-down *apo*-Rpn10 or Ub-Rpn10. Bound fractions were analysed by SDS–PAGE followed by western blot with an α-Rpn10 antibody. *Apo*-Rpn10 and Ub-Rpn10 inputs are indicated on the left side of the blot. SDS–PAGE analysis shows the immobilized protein inputs (right). (**c**) PCA analysis shows that Rpn10 ubiquitylation at K84 destabilizes the interaction with Rpn9 *in vivo*. Ten-fold serial dilutions of strains containing the N terminus fusions of *RPN9* (F[1,2]::*RPN9*), combined with either [F3]::*RPN10* or [F3]::*rpn10-K84R* (*RPN10* mutated at lysine 84), were plated on a rich medium lacking (control) or supplemented with 200 μg ml^−1^ methotrexate. *CDC19*::F[1,2]/*MCK1*::F[3] and *MET18*::F[1,2]/*MCK1*::F[3] fusion proteins were used as positive and negative controls, respectively. (**d**) Rpn10 ubiquitylation on purified proteasomes. *rpt6Δ* yeast strains expressing wild type or *Rpn10*-K84 mutant or *Rpn10Δ* from the native chromosomal locus were transformed with vector expressing Tap-proteinA-Rpt6. Proteasomes from the indicated strains were affinity-purified and immobilized on calmodulin resin. The immobilized proteasomes were incubated with Rpn10 ubiquitylation buffer that containing bacterial purified Ub, E1, Ubc4 and Rsp5. Bound and unbound fractions were separated on columns, and samples were resolved by SDS–PAGE, followed by western blot with the indicated antibodies. Ubiquitylated-Rpn10 was observed only in the unbound fraction.

Superimposing our determined Ub-Rpn10 structure onto the Rpn10 from four cryo-EM models of the proteasome^24^,^25^ revealed that the conjugated Ub clashes into Rpn9 (Fig. 5a). This suggests that Rpn10 ubiquitylation could lead to disassembly of the Rpn10:Rpn9 complex and promote the release of either Rpn9 or Ub-Rpn10 from the proteasome. As Rpn9 is tightly associated with the proteasome and buries nearly 3,000 Å^2^ by interacting with four neighbours, whereas Rpn10 is loosely associated and buries only ∼1,200 Å^2^ (Supplementary Fig. 8), we propose that Rpn10 ubiquitylation dissociates Ub-Rpn10, rather than Rpn9, from the proteasome. To test this hypothesis, we performed a series of *in vitro* and *in vivo* experiments. To examine whether Rpn10 ubiquitylation directly dissociates the Rpn10:Rpn9 complex without the need of additional proteasomal components, we performed an *in vitro* binding assay with bacterially purified proteins. Rpn10 partners including Rpn9, Dsk2-UbL, Ub^WT^ or Ub^L8E,I44E,V70D^ mutant were expressed and affinity-purified from *E. coli.* These immobilized proteins were then used to pull-down Rpn10 or Ub-Rpn10 (Fig. 5b). We found that Rpn9 loosely but significantly bound *apo*-Rpn10. Moreover, as our model predicts, Rpn9 did not bind Ub-Rpn10.

A recent NMR study provided high-resolution insight into the structure of Rpn9 (ref. 31). Chemical shift perturbations analysis of the Rpn10:Rpn9 interface is in agreement with our results showing that the Rpn10 surface, including E14, Y15, R17, N18 and G19, interacts with Rpn9. These data are also in agreement with the superposition of our crystal structure projected on the proteasome, as it suggests a competition of Rpn9 with the conjugated Ub on the same interface of Rpn10. As could also be predicted from the structure, no significant effect of Ub conjugation on the binding of Rpn10 to Dsk2-UbL or Ub^WT^ was observed (Fig. 5b). While the *S. pombe* Rpn10 was shown to interact with Rpn12 (ref. 19), this association is not seen in the cryo-EM structure of the *S. cerevisiae* proteasome^24^,^25^. Our experiments indicate that neither Rpn10 nor Ub-Rpn10 bind Rpn12, corroborating the EM models. This experiment suggests that the K84 conjugated Ub competes with the Rpn10:Rpn9 non-covalent interaction.

To test this hypothesis *in vivo*, we used a protein-fragment complementation assay (PCA) in yeast. The PCA is based on a split methotrexate (MTX) resistant DHFR mutant^32^,^33^. The N- and C-termini DHFR fragments were fused to the N-termini of Rpn9 and Rpn10 or an Rpn10-K84R mutant, respectively, at their native chromosomal loci. The ability of these yeast strains to confer methotrexate resistance was examined (Fig. 5c). In agreement with our model, moderate growth was observed using Rpn10-WT under restrictive conditions, indicating that Rpn10 loosely interacts with Rpn9. This finding indicates the dynamics of the Rpn10 association with Rpn9 and with the proteasomes. However, under the same restrictive conditions, a more than 100-fold diluted starter of the Rpn10-K84R mutant grew similarly to wild-type Rpn10 (Fig. 5c), indicating a significantly tighter Rpn10:Rpn9 interaction. This result indicates that ubiquitylation at K84 dissociates the Rpn9:Rpn10 complex. As Rpn9 is a permanent resident of the proteasome, these data also suggest that Rpn10 ubiquitylation releases Ub-Rpn10 from the proteasome *in vivo*.

We then examined whether Rpn10 ubiquitylation at K84 could dissociate Ub-Rpn10 from affinity-purified proteasomes (Fig. 5d). We constructed yeast strains that express wild-type Rpn10, Rpn10Δ or the Rpn10-K84R point mutant from the native chromosomal locus and transformed them with tap-Rpt6. The tap-tagged proteasomes were affinity-purified and immobilized on a calmodulin affinity matrix. We found that both Rpn10-WT and Rpn10-K84R associate with the proteasome at the same amount, indicating that the K84R mutation does not affect the Rpn10:Rpn9-binding interface (Fig. 5d bound fraction). Next, a ubiquitylation mixture containing yeast Ub, Uba1 (E1), Ubc4 (E2) and Rsp5 (E3) was added to the immobilized proteasomes, and the localization of Rpn10 and Ub-Rpn10 were monitored in the bound and the unbound proteasome fractions. Under these conditions, Rpn10 underwent ubiquitylation and that Ub-Rpn10 was found only in the unbound fraction (Fig. 5d). This finding suggests that ubiquitylation dissociated the modified protein from the immobilized proteasomes. Although it has been reported that Rpn10 undergoes Rsp5-dependent ubiquitylation on the proteasomes^7^, we cannot exclude the possibility that under the experimental conditions some unbound Rpn10 underwent ubiquitylation as well. Moreover, these experiments also showed that Ub-Rpn10 could not associate with proteasomes, hence ubiquitylation drives a change in distribution of the bound/unbound Rpn10, in favour of the unbound population. Collectively, the crystal structure and its interpretation in the context of the cryo-EM data, the *in vivo* and the *in vitro* experiments—point towards a scenario where K84 ubiquitylation dissociates Rpn10 from the proteasome due to steric clashes with Rpn9.

## Discussion

Structures of ubiquitylated proteins shed light on the interpretation mechanisms of the Ub-signal within its biological context. Until recently, however, difficulties in obtaining sufficient quantities of authentic ubiquitylated proteins have impeded such structural studies^20^,^34^,^35^. Advanced chemical biology developments have recently facilitated the crystallization and structure determination of ubiquitylated-H2B complex with the SAGA co-activator^36^. Here we employed a straightforward bacterial expression/purification system to circumvent this limitation^5^,^16^.

Many studies have focused on the proteasomal assembly of Rpn10 and other shuttle proteins (including Rad23, Dsk2 or Ddi1). On the basis of these findings, it was particularly suggested that a large amount of cytosolic Rpn10 molecules harvest and shuttle ubiquitylated proteins to the proteasomes for destruction^11^,^14^,^15^,^37^,^38^. As the shuttling model was never fully demonstrated, over the years many nuances were suggested to the model.

**Figure 6:**
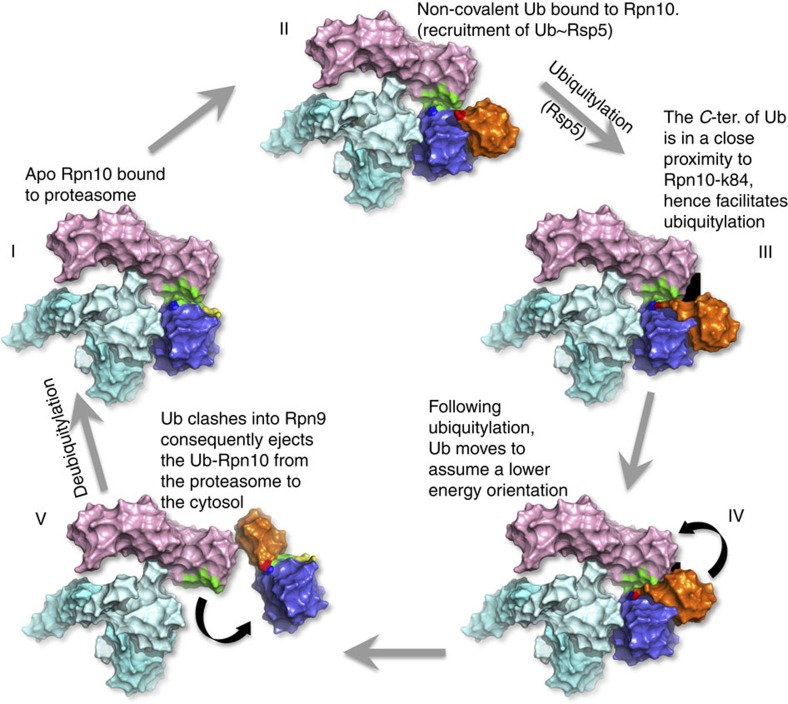
Model: ubiquitylation regulates Rpn10 dissociation from the proteasome. Schematic model for the regulation of Rpn10 by ubiquitylation: (I) *Apo*-Rpn10 (purple) is bound to the proteasome. Rpn9, pink; Rpn8, light blue; Rpn11, cyan; non-covalent Ub-binding patch, yellow; Rpn10:Rpn9 interface, green. (II) Ub∼Rsp5 interacts with the non-covalent Ub-binding patch on vWA. Ub, orange; Ub-G76, red; Rpn10-K84, blue. (III) In the non-covalent interaction, the C terminus of Ub is located in proximity to K84. (IV) Upon ubiquitylation, the Ub moiety, covalently linked to K84, assumes a lower-energy conformation (the black background is intended to facilitate perception of Ub motion). (V) The Ub moiety clashes with Rpn9 and ejects Ub-Rpn10 from the proteasome. Dissociation of Ub-Rpn10 from the proteasome was recently demonstrated by Crosas and co-workers^39^. Debiquitylation probably returns Rpn10 to its *apo*-free form and allows a next cycle of Rpn10-dependent ubiquitylated substrate degradation.

Recently, Crosas and co-workers demonstrated that Rsp5-dependent ubiquitylation of Rpn10 promotes the dissociation of the Ub-receptor from the proteasome^39^. However, the detailed structural mechanism that induces the dissociation remained unknown. Our structure provides structural insight into this highly important part of the shuttling model: the mechanism that triggers disassembly of Rpn10 from the proteasome, thereby facilitating the cyclic activity of Rpn10. We suggest that Rpn10 functions as a both proteasomal and cytosolic Ub-receptor that harvests ubiquitylated proteins for proteasomal destruction. Our model suggests that the cytosolic form of Rpn10 shuttles ubiquitylated proteins to Rpn10-free proteasomes. The Rpn10:ubiquitylated-cargo complex associates with the proteasome via direct interactions of the vWA domain with Rpn9, Rpn8 and Rpn11 and via indirect interactions of the Ub moieties with other proteasomal Ub-receptors, such as Rpn13 and/or Rpn1. Deubiquitylation, cargo unfolding, and degradation then take place, rendering the proteasome occupied with *apo*-Rpn10. This *apo*-Rpn10 can either function as a proteasomal Ub-receptor resident, or dissociate from the proteasome to allow a next cycle of cytosolic Rpn10:ubiquitylated-cargo binding and degradation. The current study elucidates a detailed structural mechanism behind the process of Rpn10 dissociation from the proteasome (Fig. 6). We suggest that substrate release allows the Rpn10 UIM domain to recruit Ub-Rsp5 and to promote self-ubiquitylation^3^. The discovered Ub-binding patch on the vWA domain directs this ubiquitylation to K84 (Figs 3, and ). Our crystallographic data imply that Ub-Rpn10 assumes at least two conformers: (i) a high-energy state in which Ub interacts with the I147 patch in a non-covalent manner and is prerequisite to ubiquitylation (Figs 2 and 3); and (ii) a low-energy state, in which the covalently linked Ub interacts with Rpn10 residues centered at E14, Y15, and R17 (Fig. 1). In order for a transition between these two states to take place, the C terminus of Ub must be positioned at a distance of <2.0 Å from the NH_2_ of Rpn10-K84. The C terminus of Ub is highly flexible and can assume multiple conformations. While retaining the general determined structure of the non-covalent Rpn10:Ub complex, we could model the C terminus of Ub in a close proximity to K84, thus allowing ubiquitylation (Fig. 6 III and Supplementary Fig. 9). As the crystal structure samples one of the energetically minima states of Ub-Rpn10, a transition from the non-covalent state to the lower-energy state is feasible (Fig. 6 III and IV). We suggest that this transition is the driving force to release Ub-Rpn10 from the proteasome (Fig. 6 V). Indeed, superimposing the Ub-Rpn10 structure onto the proteasome shows a clash of the Ub moiety with Rpn9 (Fig. 5a). This result is corroborated by our *in vivo* and *in vitro* analyses (Fig. 5b–d). Finally, we suggest that deubiquitylation regenerates *apo*-Rpn10 molecules for succeeding cycles. Ubp2 was shown to remove conjugated Ub from Rpn10 (ref. 7). Interestingly, Ubp2 forms a complex with Rsp5, demonstrating the function of these two enzymes as a molecular switch^40^.

Such a shuttling model substantially contributes to the function of the proteasome, as it allows a large number of cytosolic Rpn10 molecules to harvest ubiquitylated substrates for proteasomal degradation, thereby increasing the proteasome processivity, and may compensate for the low diffusion coefficient of such a large complex. Our model is supported by observations that, under stress conditions, where protein misfolding is induced, Rpn10 becomes essential^37^.

The presented structure provides high-resolution insight into the regulation mechanisms of Ub-receptors by coupled ubiquitylation. We suggest that ubiquitylation of Ub-receptors is vital, as it promotes cyclic activity rather than blocking their UBD(s) to inhibit them^1^,^6^. Future studies can further explore and refine this new paradigm.

## Methods

### DNA cloning and manipulations

The open reading frame of *S. cerevisiae Rpn10* (encoding residues 1–268) was amplified by PCR from pRS424-*Rpn10*. The PCR product was cloned into the SacII-NotI endonuclease restriction sites of a modified pCDF-duet vector lacking the N-terminal His_6_ tag but harbouring a maltose-binding protein (MBP) tag, followed by a PreScission protease (human rhinovirus type 14–3C protease) recognition site. The resulting vector expresses an MBP–Rpn10 fusion protein and is named pCOG31. The ubiquitylation cascade components His_6_-Ub, E1 Uba1 and E2 Ubc4 (yeast) were expressed from pGEN24, and the E3 ligase NusA-Rsp5 (yeast) was expressed from pCOG30. vWA constructs encoding 1–189 were created from pGST-Rpn10 plasmid by PCR reaction designed to delete residues 190–268. The coding sequences of proteasome subunits Rpn9 was amplified from *S. cerevisiae* DNA by PCR, digested with BamHI and EcoRI, and cloned into the pHis-parallel2 vector between the BamHI and EcoRI endonuclease restriction sites. The *Rpn12* open reading frame was amplified by PCR from the genomic DNA of *S. cerevisiae*, digested with BglII and NotI, and cloned into the pHis-parallel2 vector between the BamHI and NotI endonuclease restriction sites. Point mutations were introduced using the ExSite (Stratagene) protocols. Supplementary Table 1 summarizes all the plasmids used in this study.

### Determination of the Ub-Rpn10 structure

Ub-Rpn10 was expressed, purified, crystallized and diffracted as reported^5^,^16^. We found that the extremely thin crystals (1–3 μm) were highly sensitive to radiation damage. We therefore used the software BEST of Popov and co-workers^41^, which precisely predicted an efficient data collection strategy to achieve a full completeness data set for these C2 space group crystals, with only 95 images at oscillation steps of 1.75°. It seems that BEST was a key to determine the structure as additional images beyond the suggested strategy were found to be highly defective by radiation damage. Data were collected at the ID14-4 beamline (ESRF) at wavelength of *λ*=0.93930 Å and cryo conditions of 100 K. The structure was determined by molecular replacement using PHASER^42^, where the Rpn10-vWA domain from *S. pombe* (PDB 2X5N) and Ub (PDB 1UBQ)^43^ were used as initial search models. PHENIX auto-build was used to provide an initial model. Model building and refinement were carried out with PHENIX^44^, Refmac5 (ref. 45) and COOT^46^. The geometry parameters of the isopetide bond were restrained within the refinement process in PHENIX. The structure was validated with PROCHECK^47^ and the PDB validation tool. Statistics of Ramachandran analysis yielded 98.7% of the residues were found in the most favoured or additional allowed regions and 1.3% were found in the generously allowed regions. None of the residues were found in disallowed regions.

We found that the linker tethering the vWA domain to the UIM has an intrinsic propensity for cleavage at the beginning of the hinge. Indeed, the linker spontaneously clipped-off during crystallization. Consequently, the crystallized protein contained the vWA domain (residues 1–191) and its conjugated Ub at K84. Interestingly, the structures of *apo*-Rpn10 from *S. cerevisiae* and from *S. pombe*^19^,^25^ also present truncated forms of the protein at the same location.

### *In silico* identification of UBD

The structures E2-25kDa complex with non-covalent Ub was used as template to probe the vWA surface for a potential Ub-binding patch using SiteEngine where the Ub coordinate file was modified to have a format of small molecule compound, that is, all residues were renamed to have the same identifier^21^. However, no further refinement processes such as the FiberDock docking steps were employed. Aligned physico-chemical properties were rendered and inspected using PyMol.

### Genetic selection assays for structural characterization

*Data collection*. *E. coli* W3110 expressing the pND-Ub, pCD-Rpn10 derivatives and pGST-Rsp5 grew to logarithmic phase at 37 °C in 5 ml of LB medium supplemented with 23 μg ml^−1^ Kanamycin, 16 μg ml^−1^ Streptomycin and 33 μg ml^−1^ Ampicillin. The cultures were collected and washed with 5 ml minimal Davis. Bacterial densities were adjusted to OD_600nm_ value of 0.3. Samples (2.5 μl each) were spotted on Davis agar Petri dishes containing 10 μg ml^−1^ trimethoprim. Time-lapse of 30 min scanning took place in 26 °C incubator using a regular A4/US-letter office scanner (Epson Perfection V37)^41^.

*Image analysis*. Images were read into Fiji^43^ as a stack using ‘import -> image sequence'. The spots-densities were measured using the Time Series Analyzer V3 (Balaji J 2007; a Java ARchive ImageJ/Fiji plugin— http://rsb.info.nih.gov/ij/plugins/time-series.html). Regions of interest were specified 20 × 20 ovals and their total intensities were integrated after background subtraction. Logistic regressions of growth curves were calculated using Origin. A single parameter that describes growth efficiency was calculated as follows: the growth curve slope at the ‘half max density' was extracted and divided by its time index.

### Pull-down experiment

Rpn9, Rpn12, Dsk2-UbL domain, Ub and a Ub mutant were expressed as His_6_ fusions and were affinity-purified on an Ni^2+^-sepharose. The immobilized proteins were washed with buffer 50 mM Tris 150 Mm NaCl, 20 mM imidazole, pH 7.4, and were then washed twice with pull-down binding buffer: 25 mM HEPES, 75 mM NaCl, 0.25% Triton X-100, 1 mM dithiothreitol (DTT; pH 7.4). Aliquots of 15 μl Ni^2+^-beads with the indicated bound proteins were prepared. Next, 42 μl of 2.8 mg ml^−1^ purified Rpn10 or Ub-Rpn10 in pull-down buffer were added to the aliquots, and the mixtures were incubated for 3 h at 4 °C. The samples were centrifuged (500*g*, 3 min), and the unbound fractions were removed. The immobilized proteins were washed six times with 1 ml of pull-down buffer. Finally, 40 μl of gel-loading buffer was added and the samples were heated to 90 °C for 5 min. The samples were analysed by SDS–PAGE followed by western blot analysis with rabbit α-*S.c.*-Rpn10 antibody. The protein bands were transferred to a nitrocellulose membrane and incubated for 1 h at room temperature with mild shaking with Odyssey blocking buffer (LI-COR) diluted 1:1 in PBS buffer, and infrared dye coupled goat α-rabbit secondary antibody (1:12,000, LI-COR). Following incubation, the antibody was removed, and the membrane was washed extensively with PBS supplemented with 0.1% Tween 20. A final washing step was with PBS only. Scanning was performed with the Odyssey infrared imaging system (LI-COR Biosciences) in accordance with the manufacturer's instructions at 700 and 800 nm.

### *In vivo* protein–protein interaction assays

All the strains used for the PCA are isogenic to BY4741, BY4742 or BY4743. The strain expresses the *Rpn10-K84* point mutant gene constructed by homologous recombination, where the wt-*Rpn10* was replaced with the mutant to ensure identical expression environment. The growth assay was performed as described^48^. F[1,2] (N-terminal fragment) and F[3] (C-terminal fragment) fusions were generated using one-step PCR-mediated homologous recombination. Synthetic complete medium supplemented with 2% glucose (s.d.; 0.17% yeast nitrogen base, 0.5% (NH_4_)_2_SO_4_, amino acids and 4% noble agar (Difco)) was used for the growth assay. Methotrexate was supplemented to a final concentration of 200 μg ml^−1^.

### Surface plasmon resonance experiments

Experimental set-up comprised α-GST antibody immobilized on a CM5 chip according to the manufacturer's protocol (GE Healthcare). The ligands (Rpn10 derivatives) were expressed and purified as GST-fusion-proteins and captured on the chip. Free mono-Ub was the analyte. Before each experiment the ligand and the analyte proteins were subjected to size exclusion chromatography. Each measurement was taken in triplicate. The experiment comprised 90–100 s for binding, 300–350 s for dissociation. Wild-type and mutant Ub analytes were injected at a flow rate of 10 ml min^−1^ in 10 mM HEPES (pH 7.0), 150 mM NaCl and 0.005% polysorbate−20 at 25 °C. At the end of each experiment, the ligands were removed and surface regeneration was achieved by flowing 10 mM glycine–HCl (pH 2.6), followed by sequential washing steps of 1–2 min with 0.1% SDS, 10 mM NaOH. Data were processed using Biacore BIAevaluation software. A single-site-binding model was used for curve fitting of the binding data (Sigma Plot). For plotting, data were scaled such that *R*_max_=100.

### Mass spectrometry

*In gel* tryptic digestion was performed for wild-type and mutant Rpn10 proteins as described in refs 5, 49. Supplementary Fig 10 shows a representative uncropped SDS–PAGE used in this study. The resulting peptides were analysed by LC-MS/MS using the Q-Exactive mass spectrometer (Thermo Scientific, Bremen, Germany) coupled online with a RSLC nano UHPLC (Ultimate 3,000, Thermo Scientific, Bremen, Germany). Samples were loaded on a 100 μm, 2 cm nanoviper pepmap100 trap column in 2% acetonitrile, 0.1% formic acid, at a flow rate of 15 μl min^−1^. Peptides were eluted and separated at a flow rate of 300 μl min^−1^ on Thermo RSLC nanocolumn 75 μm × 15 cm, pepmap100 C18, 3 μm 100 Å pore size, with a linear acetonitrile gradient from 2 to 24% in 0.1% formic acid for 9 min followed by a linear increase to 32% acetonitrile in 0.1% formic acid over 1 min and additional increase up to 80% acetonitrile in 0.1% formic acid over 5 min, followed by reduction of acetonitrile back to 2% and re-equilibration. The eluent was nebulized and ionized using the Thermo nano electrospray source with a distal coated fused silica emitter (New Objective, Woburn, MA, USA), with a capillary voltage of 1.8–2.2 kV. The Q-Exactive instrument was operated in the data-dependent mode to automatically switch between full-scan MS and MS/MS acquisition. Survey full-scan MS spectra (*m*/*z* 375–1,850) were acquired in the Orbitrap with 70,000 resolution (*m*/*z* 200) after accumulation of ions to a 3 × 10^6^ target value with maximum injection time of 120 ms. The 10 most intense multiply charged ions (2≤*z*≤6) were sequentially isolated and fragmented in the octopole collision cell by higher-energy collisional dissociation, with a fixed injection time of 60 ms 17,500 resolution and AGC target of 1 × 10^5^ counts. A 2.7 Da isolation width was chosen. Underfill ratio was at 10% and dynamic exclusion was set to 10 s. Typical mass spectrometric conditions were as follows: spray voltage, 2 kV; no sheath and auxiliary gas flow; heated capillary temperature, 275 °C; normalized higher-energy collisional dissociation collision energy 27%. The mass spectrometry data were analysed using the trans proteomic pipeline (TPP) Version 4.6.3 (ref. 50) TPP-processed centroid fragment peak lists in mzML format were searched against a database composed of yeast and *E. coli* proteins (Uniprot) and human ubiquitin supplemented with their corresponding decoy sequences (as described in http://www.matrixscience.com/help/decoy_help.html). The database searches were performed using X! Tandem with high-resolution k-score plugin through the TPP. Search parameters comprised: trypsin cleavage specificity with two missed cleavage, cysteine carbamidomethyl as fixed modification, methaionine oxidation, and protein N-terminal acetylation and lysine GG as variable modifications, peptide tolerance and MS/MS tolerance were set at 20 p.p.m. X! Tandem refinement comprised: semi-style cleavages and variable lysine GG modification. Peptide and protein lists were generated following Peptide Prophet and Protein Prophet analysis using protein FDR of <1%.

### Proteasomes purification and Rpn10 ubiquitylation

Yeast strains expressing TAP-tagged Rpn6 (Mata his3-D200 lys2-801 leu2-3,112 trp1-1 ura3-52, rpt6::HIS3, rpn10::KanMX6, (TAP-RPT6: LEU2) were transformed with *WT-Rpn10*, *Rpn10-K84R* or empty (*Rpn10Δ*) vectors and grown on media lacking Uracil (-Ura). Cells were harvested by centrifugation (5 min, 4,000 r.p.m., 4 °C), the pellet was re-suspended in binding buffer (50 mM Tris 7.4, 200 mM NaCl, 10 mM MgCl_2_, 2 mM CaCl_2_, 10% glycerol, 1 mM ATP and 1 mM DTT), and the cells were lysed with glass beads. The soluble fraction was separated by centrifugation and the proteasomes were affinity-purified on calmodulin resin. Immobilized TAP-Rpt6 proteasomes (108 μl) were then incubated for 60 min, at 30 °C, with or without 28 μl of Rpn10 ubiquitylation buffer (0.9 μg His_6_-Uba1, 2.25 μg His_6_-Ubc4, 6 μg MBP-Rsp5, 12.5 μg Ub and 50 mM Tris (pH 7.5), 200 mM NaCl, 1 mM DTT, 10 μM ATP). Samples were loaded on a column and the unbound fraction was collected. The resin-bound fraction was washed three times with 0.5 ml binding buffer and unbound material was removed followed centrifugation (500*g*, 4 °C, 2 min). Gel-loading buffer was added, the samples were incubated at 90 °C for 5 min, and resolved by 13.5% SDS–PAGE. Finally, the gels were visualized by western blot analysis with anti-Rpn10 antibody followed by mouse anti-rabbit labeled secondary antibody.

### Data availability

The coordinates and structure factors have been deposited with the Protein Data Bank under the accession number 5LN1. The mass spectrometry proteomics data have been deposited to the ProteomeXchange Consortium via the PRIDE partner repository with the data set identifier PXD004761. The additional data that support the findings of this study are available from the corresponding author upon request.

## Additional information

**How to cite this article:** Keren-Kaplan, T. *et al*. Structure of ubiquitylated-Rpn10 provides insight into its autoregulation mechanism. *Nat. Commun.* **7,** 12960 doi: 10.1038/ncomms12960 (2016).

## Supplementary Material

Supplementary Information Supplementary Figures 1 - 10 and Supplementary Table 1

Supplementary Movie 1 Time_lapse growth movie under selective conditions

Supplementary Movie 2 Time_lapse growth movie under non-selective conditions

## Figures and Tables

**Table 1:** Data collection and refinement statistics.

*Data collection*	
Space group	C 1 2 1
Cell dimensions	
*a*, *b*, *c* (Å)	107.29, 49.70, 81.33
α, β, γ (°)	90.00, 130.55, 90.00
Resolution (Å)*	53.64–3.14 (3.31–3.14)
*R*merge	0.084 (0.225)
*I*/σ*I*	10.2 (5.2)
Completeness (%)	99.8 (99.9)
Redundancy	3.2 (3.4)
	
*Refinement*	
Resolution (Å)	53.64–3.14 (3.31–3.14)
No. of reflections	18,985 (2,875)
No. unique reflections	5,824 (857)
*R*_work_/*R*_free_	0.1926/0.2479
No. of atoms	2,069
Protein	2,069
Ligand/ion	None
Water	None
	
*B*-factors	
Protein	44.58
Ligand/ion	NA
Water	NA
	
*R.m.s. deviations*	
Bond lengths (Å)	0.010
Bond angles (°)	1.41

^*^Highest-resolution shell (3.31–3.14 Å) is shown in parenthesis.

## References

[b1] Hurley J. H., Lee S. & Prag G. Ubiquitin-binding domains. Biochem. J. 399, 361–372 (2006). 17034365 10.1042/BJ20061138 PMC1615911

[b2] Komander D. & Rape M. The ubiquitin code. Annu. Rev. Biochem. 81, 203–229 (2012). 22524316 10.1146/annurev-biochem-060310-170328

[b3] Polo S. . A single motif responsible for ubiquitin recognition and monoubiquitination in endocytic proteins. Nature 416, 451–455 (2002). 11919637 10.1038/416451a

[b4] Shih S. C. . A ubiquitin-binding motif required for intramolecular monoubiquitylation, the CUE domain. EMBO J. 22, 1273–1281 (2003). 12628920 10.1093/emboj/cdg140 PMC151082

[b5] Keren-Kaplan T. . Synthetic biology approach to reconstituting the ubiquitylation cascade in bacteria. EMBO J. 31, 378–390 (2012). 22081111 10.1038/emboj.2011.397 PMC3261559

[b6] Hoeller D. . Regulation of ubiquitin-binding proteins by monoubiquitination. Nat. Cell Biol. 8, 163–169 (2006). 16429130 10.1038/ncb1354

[b7] Isasa M. . Monoubiquitination of RPN10 regulates substrate recruitment to the proteasome. Mol. Cell 38, 733–745 (2010). 20542005 10.1016/j.molcel.2010.05.001 PMC3282119

[b8] van Nocker S. . The multiubiquitin-chain-binding protein Mcb1 is a component of the 26S proteasome in *Saccharomyces cerevisiae* and plays a nonessential, substrate-specific role in protein turnover. Mol. Cell Biol. 16, 6020–6028 (1996). 8887631 10.1128/mcb.16.11.6020 PMC231604

[b9] Ferrell K., Deveraux Q., van Nocker S. & Rechsteiner M. Molecular cloning and expression of a multiubiquitin chain binding subunit of the human 26S protease. FEBS Lett. 381, 143–148 (1996). 8641424 10.1016/0014-5793(96)00101-9

[b10] Haracska L. & Udvardy A. Cloning and sequencing a non-ATPase subunit of the regulatory complex of the Drosophila 26S protease. Eur. J. Biochem. 231, 720–725 (1995). 7649173 10.1111/j.1432-1033.1995.tb20753.x

[b11] Matiuhin Y. . Extraproteasomal Rpn10 restricts access of the polyubiquitin-binding protein Dsk2 to proteasome. Mol. Cell 32, 415–425 (2008). 18995839 10.1016/j.molcel.2008.10.011 PMC2643056

[b12] Deveraux Q., van Nocker S., Mahaffey D., Vierstra R. & Rechsteiner M. Inhibition of ubiquitin-mediated proteolysis by the Arabidopsis 26S protease subunit S5a. J. Biol. Chem. 270, 29660–29663 (1995). 8530351 10.1074/jbc.270.50.29660

[b13] Zhang D. . Together, Rpn10 and Dsk2 can serve as a polyubiquitin chain-length sensor. Mol. Cell 36, 1018–1033 (2009). 20064467 10.1016/j.molcel.2009.11.012 PMC2807407

[b14] Lipinszki Z. . Developmental-stage-specific regulation of the polyubiquitin receptors in *Drosophila melanogaster*. J. Cell Sci. 122, 3083–3092 (2009). 19654212 10.1242/jcs.049049

[b15] Hamazaki J. . Rpn10-mediated degradation of ubiquitinated proteins is essential for mouse development. Mol. Cell Biol. 27, 6629–6638 (2007). 17646385 10.1128/MCB.00509-07 PMC2099239

[b16] Keren-Kaplan T. & Prag G. Purification and crystallization of mono-ubiquitylated ubiquitin receptor Rpn10. Acta Crystallogr. Sect. F Struct. Biol. Cryst. Commun. 68, 1120–1123 (2012). 10.1107/S1744309112034331 PMC3433213 22949210

[b17] Hodel A., Kim S.-H. & Brunger A. T. Model bias in macromolecular crystal structures. Acta Crystallogr Sect. A 48, 851–858 (1992).

[b18] Prag G. . Mechanism of ubiquitin recognition by the CUE domain of Vps9p. Cell 113, 609–620 (2003). 12787502 10.1016/s0092-8674(03)00364-7

[b19] Riedinger C. . Structure of Rpn10 and its interactions with polyubiquitin chains and the proteasome subunit Rpn12. J. Biol. Chem. 285, 33992–34003 (2010). 20739285 10.1074/jbc.M110.134510 PMC2962499

[b20] Dikic I., Wakatsuki S. & Walters K. J. Ubiquitin-binding domains—from structures to functions. Nat. Rev. Mol. Cell Biol. 10, 659–671 (2009). 19773779 10.1038/nrm2767 PMC7359374

[b21] Keren-Kaplan T. . Structure-based in silico identification of ubiquitin-binding domains provides insights into the ALIX-V:ubiquitin complex and retrovirus budding. EMBO J. 32, 538–551 (2013). 23361315 10.1038/emboj.2013.4 PMC3579145

[b22] Alam S. L. . Ubiquitin interactions of NZF zinc fingers. EMBO J. 23, 1411–1421 (2004). 15029239 10.1038/sj.emboj.7600114 PMC391057

[b23] Chothia C. The nature of the accessible and buried surfaces in proteins. J. Mol. Biol. 105, 1–12 (1976). 994183 10.1016/0022-2836(76)90191-1

[b24] Unverdorben P. . Deep classification of a large cryo-EM dataset defines the conformational landscape of the 26S proteasome. Proc. Natl Acad. Sci. USA 111, 5544–5549 (2014). 24706844 10.1073/pnas.1403409111 PMC3992697

[b25] Beck F. . Near-atomic resolution structural model of the yeast 26S proteasome. Proc. Natl Acad. Sci. USA 109, 14870–14875 (2012). 22927375 10.1073/pnas.1213333109 PMC3443124

[b26] Levin-Kravets O. . A bacterial genetic selection system for ubiquitylation cascade discovery. Nature Methods DOI:10.1038/NMETH.4003 (2016). 10.1038/nmeth.4003 27694912

[b27] Lu J. Y. . Functional dissection of a HECT ubiquitin E3 ligase. Mol. Cell Proteomics 7, 35–45 (2008). 17951556 10.1074/mcp.M700353-MCP200 PMC2861892

[b28] Sakata E. . Localization of the proteasomal ubiquitin receptors Rpn10 and Rpn13 by electron cryomicroscopy. Proc. Natl Acad. Sci. USA 109, 1479–1484 (2012). 22215586 10.1073/pnas.1119394109 PMC3277190

[b29] Lasker K. . Molecular architecture of the 26S proteasome holocomplex determined by an integrative approach. Proc. Natl Acad. Sci. USA 109, 1380–1387 (2012). 22307589 10.1073/pnas.1120559109 PMC3277140

[b30] da Fonseca P. C., He J. & Morris E. P. Molecular model of the human 26S proteasome. Mol. Cell 46, 54–66 (2012). 22500737 10.1016/j.molcel.2012.03.026

[b31] Hu Y., Wu Y., Li Q., Zhang W. & Jin C. Solution structure of yeast Rpn9: insights for proteasome lid assembly. J. Biol. Chem. 290, 6878–6889 (2015). 25631053 10.1074/jbc.M114.626762 PMC4358113

[b32] Pelletier J. N., Campbell-Valois F. X. & Michnick S. W. Oligomerization domain-directed reassembly of active dihydrofolate reductase from rationally designed fragments. Proc. Natl Acad. Sci. USA 95, 12141–12146 (1998). 9770453 10.1073/pnas.95.21.12141 PMC22798

[b33] Lev I. . Reverse PCA, a systematic approach for identifying genes important for the physical interaction between protein pairs. PLoS Genet. 9, e1003838 (2013). 24130505 10.1371/journal.pgen.1003838 PMC3794912

[b34] Williamson A., Werner A. & Rape M. The Colossus of ubiquitylation: decrypting a cellular code. Mol. Cell 49, 591–600 (2013). 23438855 10.1016/j.molcel.2013.01.028 PMC3589588

[b35] Faggiano S. & Pastore A. The challenge of producing ubiquitinated proteins for structural studies. Cell 3, 639–656 (2014). 10.3390/cells3020639 PMC4092866 24926903

[b36] Morgan M. T. . Structural basis for histone H2B deubiquitination by the SAGA DUB module. Science 351, 725–728 (2016). 26912860 10.1126/science.aac5681 PMC4863942

[b37] Fu H. . Multiubiquitin chain binding and protein degradation are mediated by distinct domains within the 26S proteasome subunit Mcb1. J. Biol. Chem. 273, 1970–1981 (1998). 9442033 10.1074/jbc.273.4.1970

[b38] Tayama Y., Kawahara H., Minami R., Shimada M. & Yokosawa H. Association of Rpn10 with high molecular weight complex is enhanced during retinoic acid-induced differentiation of neuroblastoma cells. Mol. Cell Biochem. 306, 53–57 (2007). 17668154 10.1007/s11010-007-9553-z

[b39] Zuin A. . Rpn10 monoubiquitination orchestrates the association of the ubiquilin-type DSK2 Receptor with the proteasome. Biochem J. 472, 353–365 (2015). 26450923 10.1042/BJ20150609

[b40] Kee Y., Lyon N. & Huibregtse J. M. The Rsp5 ubiquitin ligase is coupled to and antagonized by the Ubp2 deubiquitinating enzyme. EMBO J. 24, 2414–2424 (2005). 15933713 10.1038/sj.emboj.7600710 PMC1173151

[b41] Bourenkov G. P. & Popov A. N. Optimization of data collection taking radiation damage into account. Acta Crystallogr. D Biol. Crystallogr. 66, 409–419 (2010). 20382994 10.1107/S0907444909054961 PMC2852305

[b42] McCoy A. J. . Phaser crystallographic software. J. Appl. Crystallogr. 40, 658–674 (2007). 19461840 10.1107/S0021889807021206 PMC2483472

[b43] Vijay-Kumar S., Bugg C. E. & Cook W. J. Structure of ubiquitin refined at 1.8Å resolution. J. Mol. Biol. 194, 531–544 (1987). 3041007 10.1016/0022-2836(87)90679-6

[b44] van Wijk S. J. . Fluorescence-based sensors to monitor localization and functions of linear and K63-linked ubiquitin chains in cells. Mol. Cell 47, 797–809 (2012). 22819327 10.1016/j.molcel.2012.06.017 PMC3714537

[b45] Murshudov G. N., Vagin A. A. & Dodson E. J. Refinement of macromolecular structures by the maximum-likelihood method. Acta Crystallogr. D Biol. Crystallogr. 53, 240–255 (1997). 15299926 10.1107/S0907444996012255

[b46] Emsley P. & Cowtan K. Coot: model-building tools for molecular graphics. Acta Crystallogr. D Biol. Crystallogr. 60, 2126–2132 (2004). 15572765 10.1107/S0907444904019158

[b47] Laskowski R. A., Macarthur M. W., Moss D. S. & Thornton J. M. PROCHECK: a program to check the stereochemical quality of protein structures. J. Appl. Cryst. 26, 283–291 (1993).

[b48] Lev I. . Reverse PCA, a systematic approach for identifying genes important for the physical interaction between protein pairs. PLoS Genet. 9, e1003838 (2013). 24130505 10.1371/journal.pgen.1003838 PMC3794912

[b49] Ziv I. . A perturbed ubiquitin landscape distinguishes between ubiquitin in trafficking and in proteolysis. Mol. Cell Proteomics 10, M111.009753 (2011). 10.1074/mcp.M111.009753 PMC3098606 21427232

[b50] Keller A., Eng J., Zhang N., Li X. J. & Aebersold R. A uniform proteomics MS/MS analysis platform utilizing open XML file formats. Mol. Syst. Biol. 1, 2005.0017 (2005). 10.1038/msb4100024 PMC1681455 16729052

