# Supplementary material for: Structure of ubiquitylated-Rpn10 provides insight into its autoregulation mechanism
Source: Nat Commun. 2016 Oct 4;7:12960. doi: 10.1038/ncomms12960 (PMC5059453; doi:10.1038/ncomms12960)
Supplement: Supplementary Information — Supplementary Figures 1 - 10 and Supplementary Table 1 [file ncomms12960-s1.pdf]

```
                    ○
                 ◆◆ ◆◆◆
Sc  MVLEATVLVIDNSEYSRNGDFPRTRFEAQIDSVEFIFQAKRNSNPENTVGLISGAGANPR 60
Sp  MVLEATMILIDNSEWMINGDYIPTRFEAQKDTVHMIFNQKINDNPENMCGLMTIGDNSPQ 60
Hs  MVLESTMVCVDNSEYMRNGDFLPTRLQAQQDAVNIVCHSKTRSNPENNVGLITLAN-DCE 59
Mm  MVLESTMVCVDNSEYMRNGDFLPTRLQAQQDAVNIVCHSKTRSNPENNVGLITLAN-DCE 59
Dr  MVLESTMVCVDNSEYMRNGDFLPTRLQAQQDAVNIICHSKTRSNPENNVGLITMAN-NCE 59
    ****:*:: :****:  ***:  **::** *:*.:: : * ..****  **:: .. . .
                         ○ ★
                        ◆◆ ◆◆
Sc  VLSTFTAEFGKILAGLHDTQIEGKLHMATALQIAQLTLKHRQNKVQHQRIVAFVCSPISD 120
Sp  VLSTLTRDYGKFLSAMHDLPVRGNAKFGDGIQIAQLALKHRENKIQRQRIVAFVGSPIVE 120
Hs  VLTTLTPDTGRILSKLHTVQPKGKITFCTGIRVAHLALKHRQGKNHKMRIIAFVGSPVED 119
Mm  VLTTLTPDTGRILSKLHTVQPKGKITFCTGIRVAHLALKHRQGKNHKMRIIAFVGSPVED 119
Dr  VLTTLTPDTGRILSKLHAVQPRGVISFCTGIRVAHLALKHRQGKNHKMRIIAFVGSPVED 119
    **:*:* : *::*: :*    .*   :  .:::*:*:****:.* :: **:*** **: :
                                ★ ★                       ★ ★
                                ■■■     ■                ■■ ■■
Sc  SRDELIRLAKTLKKNNVAVDIINFGE--IEQNTELLDEFIAAVNNPQEETSHLLTVTPGP 178
Sp  DEKNLIRLAKRMKKNNVAIDIIHIGE--L-QNESALQHFIDAANSSDSC--HLVSIPPSP 175
Hs  NEKDLVKLAKRLKKEKVNVDIINFGEEEV--NTEKLTAFVNTLNGKDGTGSHLVTVPPGP 177
Mm  NEKDLVKLAKRLKKEKVNVDIINFGEEEV--NTEKLTAFVNTLNGKDGTGSHLVTVPPGP 177
Dr  QEKDLVKMAKRLKKEKVSVDIINFGEEEV--NTEKLTVFVNTLNGKEGVGSHLVTVPPGP 177
    ...:*:::** :**::* :***::**  :  *.  *. *:  .*  .  ..**:::.*:*
              ■
Sc  RLLYENIASSPIILEEGSSGMGAFGGSGGDSDANGTFMDFGVDPSMDPELAMALRLSMEE 238
Sp  QLLSDLVNQSPI-------GQGVVASQNQ--------FEYGVDPNLDVELALALELSMAE 220
Hs  SLADALIS-SPILAGEG----GAMLGLGASD------FEFGVDPSADPELALALRVSMEE 226
Mm  SLADALIS-SPILAGEG----GAMLGLGASD------FEFGVDPSADPELALALRVSMEE 226
Dr  SLADALLS-SPIMAGEG----GTIMGLGASD------FEFGVDPSADPELALALRVSMEE 226
     *    :  ***         *.. . .         :::****. * ***:**.:** *

Sc  EQQRQERLRQQQQQ-------------------------------------DQPEQSE 260
Sp  ERARQEVAAQKS-----------------------------------------SEETE 237
Hs  QRQRQEEEARRAAAASAAEAGIATTGTEGERDSDDALLKMTISQQEFGRTGLPDLSSMTE 286
Mm  QRQRQEEEARRAAAASAAEAGIATPGTEGERDSDDALLKMTINQQEFGRPGLPDLSSMTE 286
Dr  QRQRQEEEARRAAVASAADAGVSSPTAD---ESENALLKMST------APALPDFSRMTE 277
    :: ***   ::                                            .  :*

Sc  QPEQHQDK---------------------------------------------------- 268
Sp  DKKMQE------------------------------------------------------ 243
Hs  EEQIAYAMQMSLQGAEFGQAESADIDASSAMDTSEPAKE-EDDYDVMQDPEFLQSVLENL 345
Mm  EEQIAYAMQMSLQGTEFSQ-ESADMDASSAMDTSDPVKE-EDDYDVMQDPEFLQSVLENL 344
Dr  DEQIAYALQMSMQGGEFGGSEAMDVDTAAAAAESEAPKEDEEDYDVMQDPEFLQSVLENL 337
    : :

Sc  ----------------------------------- 268
Sp  ----------------------------------- 243
Hs  PGVDPNNEAIRNAMGSLASQATKDGKKDKKEEDKK 380
Mm  PGVDPNNAAIRSVMGALASQATKDGKNDKKEEEKK 379
Dr  PGVDPNNEAIRNAMGSLASQNRTKPPEGKKEDEKK 372
```

Supplementary Figure 1. Multiple alignment of Rpn10 proteins from common organism models. Conservation is shown at the bottom of the alignment: (*) Full conservation, (:) Strongly similar residues and (.) Weakly similar residues. Symbol codes: (★) Mutated residue at the Ub:VWA non- covalent binding patch; (○) Structurally possible compensatory residue in *S. pombe* at the Ub:VWA non-covalent binding patch; (◆) Interacting residue at the Ub:VWA covalent bond area; (■) Interacting residue at the Ub:VWA non-covalent binding patch. Organism names: Sc, *Saccharomyces cerevisiae*; Sp, *Schizosaccharomyces pombe*; Hs, *Homo sapiens*; Mm, *Mus musculus*; Dr, *Danio rerio*.

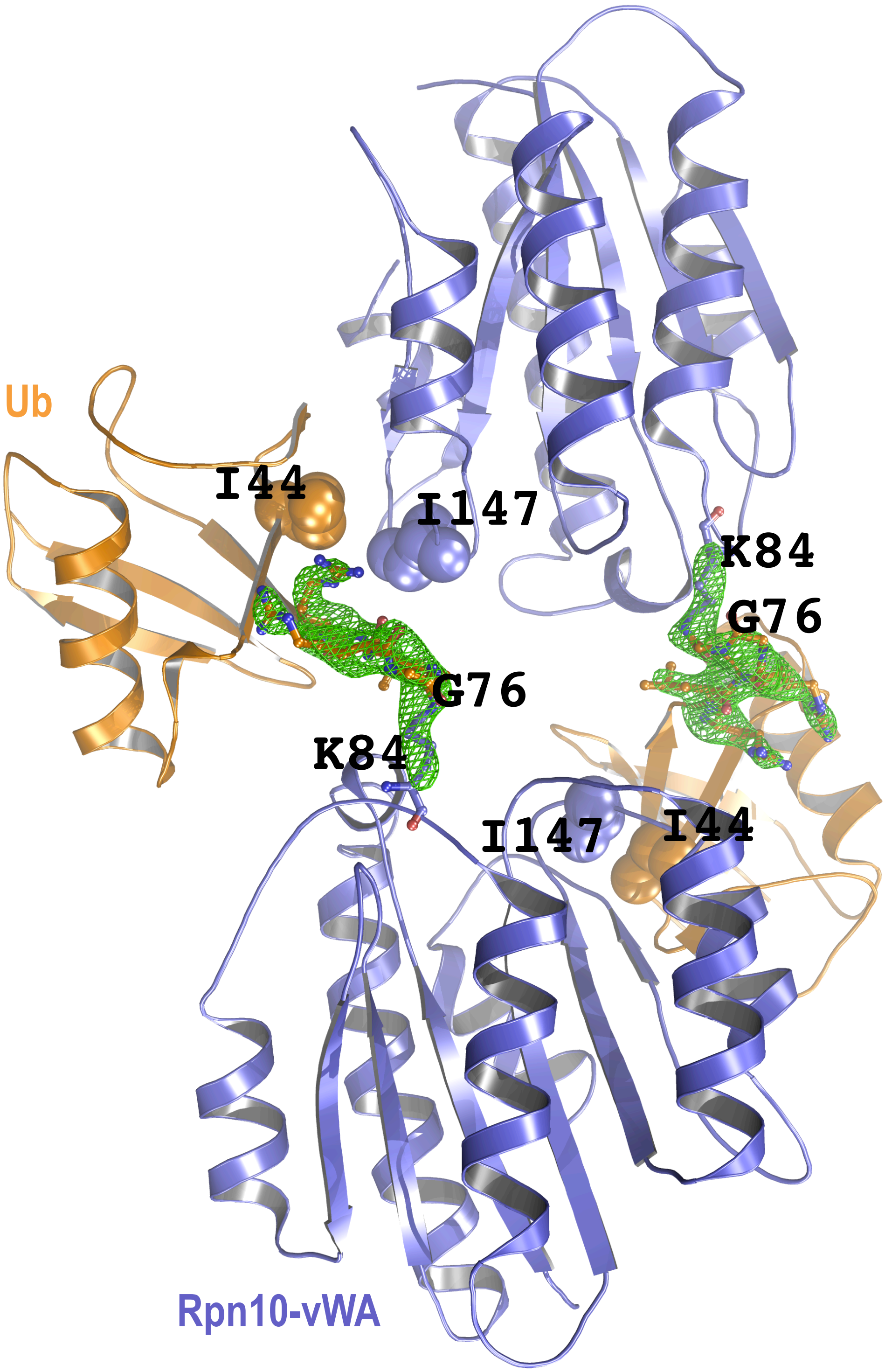

Supplementary Figure 2. Assessment of model biased by simulated annealing omit map. A simulated-annealing refinement of partial structure in which the Ub C-terminus segment [(residues R72-G76) and the residue atoms of K84 (Cβ, Cγ, Cδ, Cε and Nζ) were omitted], was performed[16]. σ_A-weighted *mFo-DFc* electron density omit-map was calculated and presented in 3σ level. The last refined model is presented.

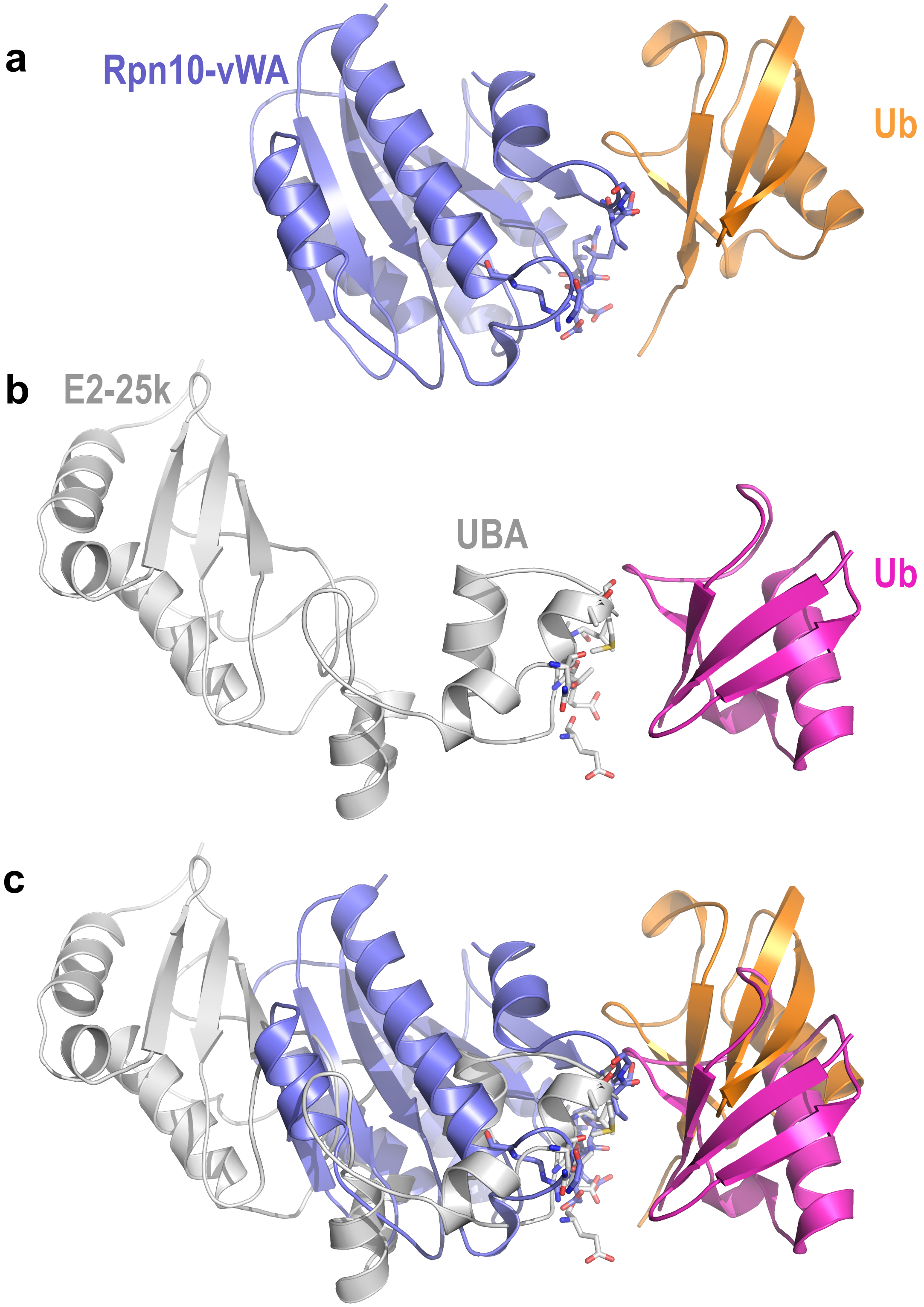

Supplementary Figure 3. Structure-based *in silico* procedure for identification of Ub-binding domains highlights the Ub binding patch on Rpn10-vWA domain. Using the structure of E2-25kDa complex with Ub (PDB 3K9P) as searching probe for Ub-binding sites on the vWA structure yielded a very similar binding mode to the one found in the non-covalent structure at the crystal lattice. a. shows the non-covalent structure of vWA:Ub. b. shows the non-covalent complex of E2-25k with Ub. c. Shows superimposition of the structures based on the *in silico* procedure (see Supplementary Fig. S3 for molecular details)

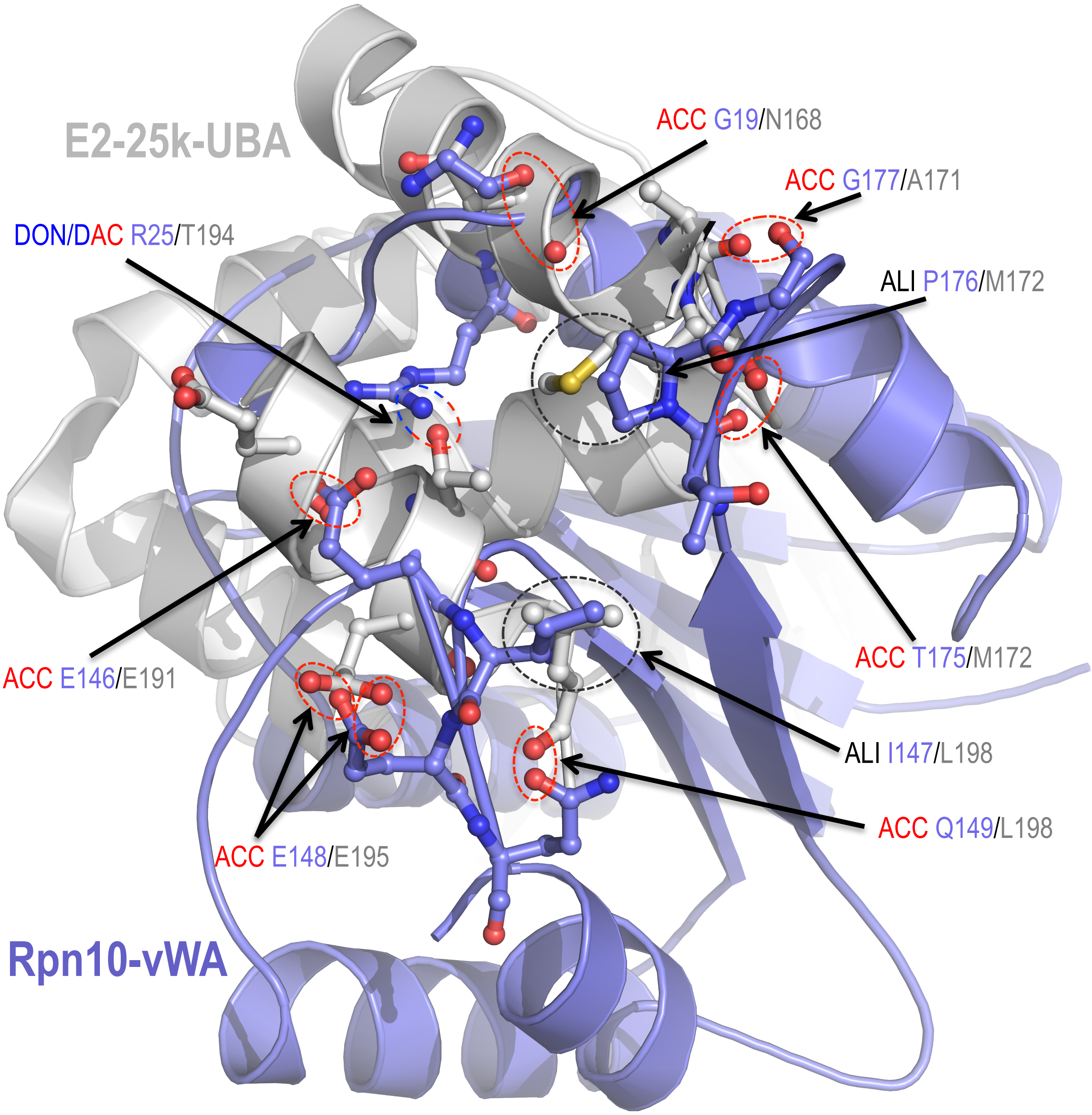

Rigid Transformation: -1.44662 -0.362251 0.387384 -2.43399 -20.2258 41.095

Similarity Score: 4187.62 rmsd: 0.692565 Match Size: 10

Chain.ID	Amino Acid	Property	Source	Chain.ID	Amino Acid	Property	Source	Dist.	Conserved AA
A.19	Gly	ACC	b	A.168	Asn	ACC	b	2.6	
A.25	Arg	DON	s	A.194	Thr	DAC	b	1.1	
A.146	Glu	ACC	s	A.191	Glu	ACC	b	1.3	*
A.147	Ile	ALI	s	A.198	Leu	ALI	b	0.39	
A.148	Glu	ACC	s	A.195	Glu	ACC	b	1.7	*
A.148	Glu	ACC	s	A.195	Glu	ACC	b	1.1	*
A.149	Gln	ACC	s	A.198	Leu	ACC	b	1	
A.175	Thr	ACC	b	A.172	Met	ACC	b	1.4	
A.176	Pro	ALI	s	A.172	Met	ALI	b	1.4	
A.177	Gly	ACC	b	A.171	Ala	ACC	b	1.3	

Supplementary Figure 4. Detailed *in silico* analysis highlights the Ub binding patch on vWA. Using the structure of E2-25kDa complex with Ub (PDB 3K9P) as searching probe for Ub binding sites on the Rpn10-vWA domain yielded a very similar binding mode to the one found in the non-covalent vWA:Ub crystal structure. Superimposition of the structures based on the *in silico* procedure highlights the aligned physico-chemical properties. The table provides an alphanumeric presentation of the aligned physico-chemical properties found in vWA with respect to the UBA domain of the E2-25kDa. ACC, acceptor; DON, donor; DAC, donor/acceptor; and ALI, aliphatic.

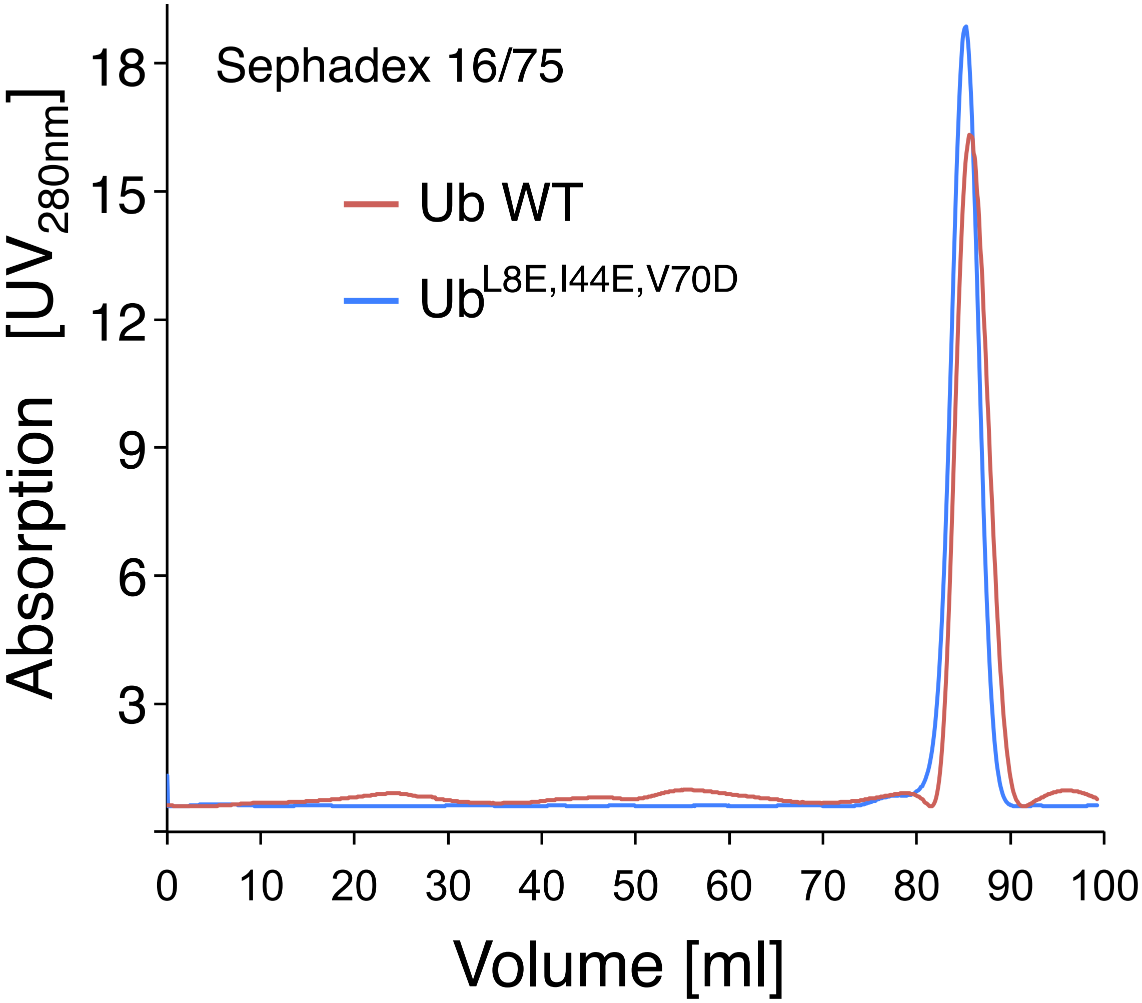

Supplementary Figure 5. The ubiquitin triple mutant at the hydrophobic patch is properly fold. Size Exclusion Chromatography (SEC) analyses of wild (red) type and L8E,I44E,V70D triple mutant (blue) of Ub proteins are shown. Purified proteins were concentrated to about 15 mg/ml in 150 mM NaCl 50 mM Tris-HCl pH 8.0. Five ml of each protein were loaded onto SEC column (Sephadex 16/75) and run at 1 ml/min using Akta Prim. The monitored optical density absorption at $UV_{280\ nm}$ is shown.

a

Ub-K84

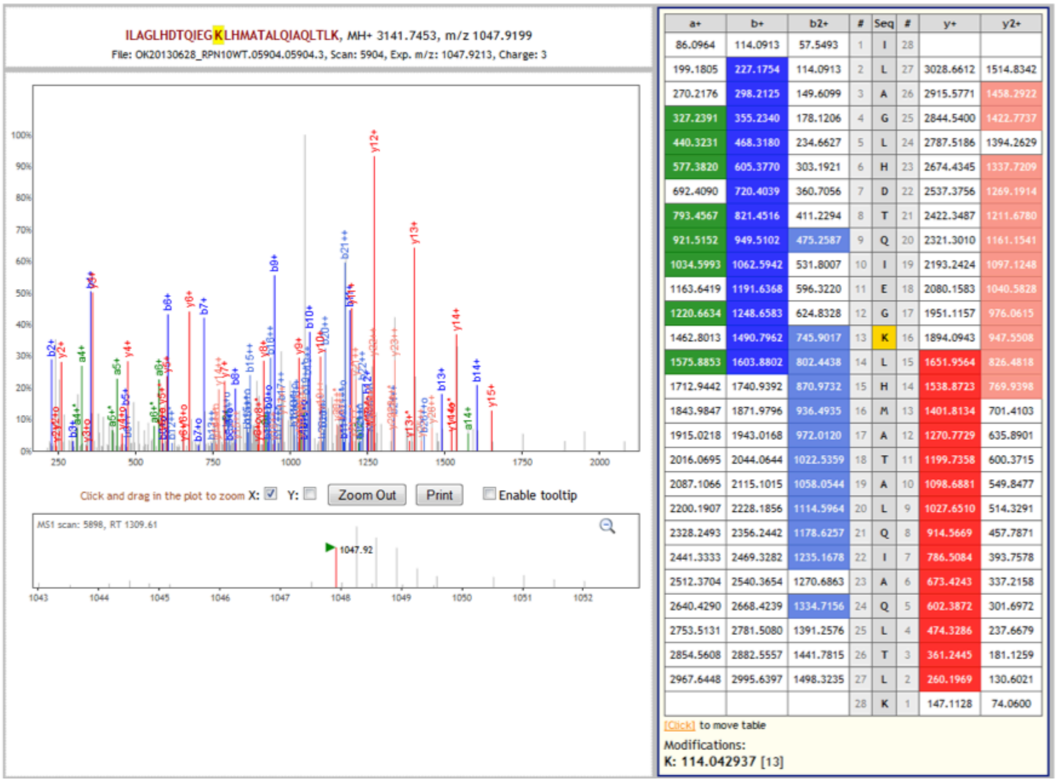

a+	b+	b2+	#	Seq	#	y+	y2+
86.0964	114.0913	57.5493	1	I	28		
199.1805	227.1754	114.0913	2	L	27	3028.6612	1514.8342
270.2176	298.2125	149.6099	3	A	26	2915.5771	1458.2922
327.2391	355.2340	178.1206	4	G	25	2844.5400	1422.7737
440.3231	468.3180	234.6627	5	L	24	2787.5186	1394.2629
577.3820	605.3770	303.1921	6	H	23	2674.4345	1337.7209
692.4090	720.4039	360.7056	7	D	22	2537.3756	1269.1914
793.4567	821.4516	411.2294	8	T	21	2422.3487	1211.6780
921.5152	949.5102	475.2587	9	Q	20	2321.3010	1161.1541
1034.5993	1062.5942	531.8007	10	I	19	2193.2424	1097.1248
1163.6419	1191.6368	596.3220	11	E	18	2080.1583	1040.5828
1220.6634	1248.6583	624.8328	12	G	17	1951.1157	976.0615
1462.8013	1490.7962	745.9017	13	K	16	1894.0943	947.5508
1575.8853	1603.8802	802.4438	14	L	15	1651.9564	826.4818
1712.9442	1740.9392	870.9732	15	H	14	1538.8723	769.9398
1843.9847	1871.9796	936.4935	16	M	13	1401.8134	701.4103
1915.0218	1943.0168	972.0120	17	A	12	1270.7729	635.8901
2016.0695	2044.0644	1022.5359	18	T	11	1199.7358	600.3715
2087.1066	2115.1015	1058.0544	19	A	10	1098.6881	549.8477
2200.1907	2228.1856	1114.5964	20	L	9	1027.6510	514.3291
2328.2493	2356.2442	1178.6257	21	Q	8	914.5669	457.7871
2441.3333	2469.3282	1235.1678	22	I	7	786.5084	393.7578
2512.3704	2540.3654	1270.6863	23	A	6	673.4243	337.2158
2640.4290	2668.4239	1334.7156	24	Q	5	602.3872	301.6972
2753.5131	2781.5080	1391.2576	25	L	4	474.3286	237.6679
2854.5608	2882.5557	1441.7815	26	T	3	361.2445	181.1259
2967.6448	2995.6397	1498.3235	27	L	2	260.1969	130.6021
			28	K	1	147.1128	74.0600

[Click] to move table

Modifications:
K: 114.042937 [13]

b

Ub-K84+Ox-M87

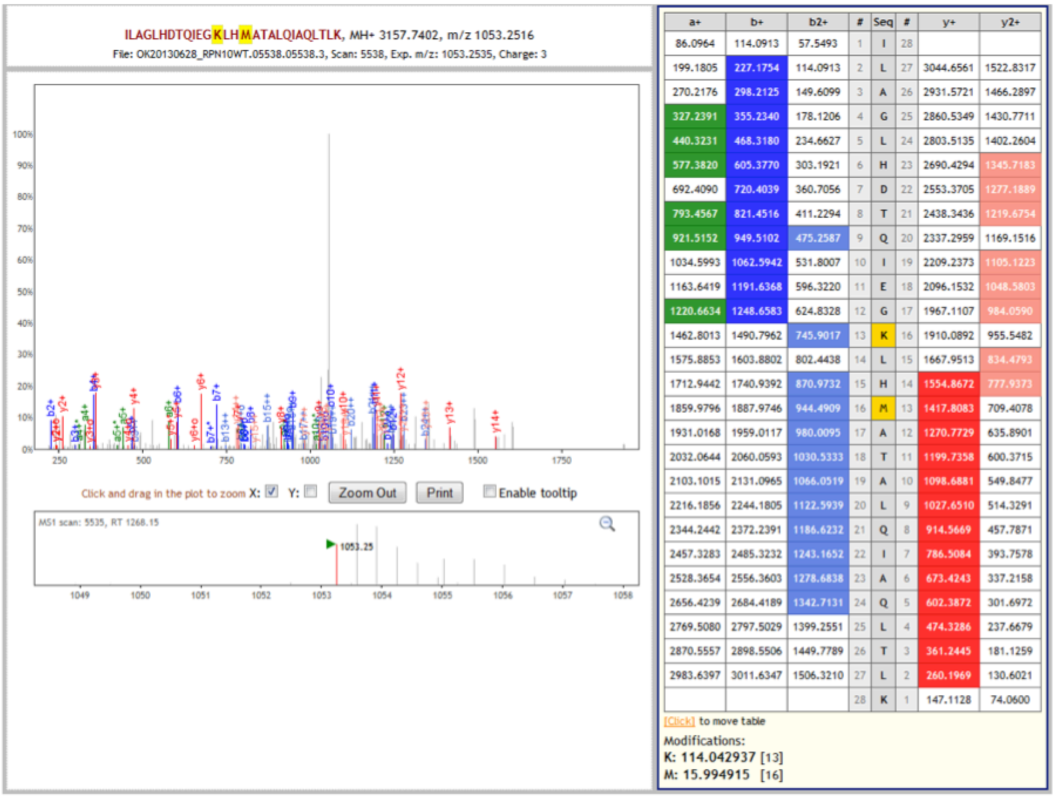

a+	b+	b2+	#	Seq	#	y+	y2+
86.0964	114.0913	57.5493	1	I	28		
199.1805	227.1754	114.0913	2	L	27	3044.6561	1522.8317
270.2176	298.2125	149.6099	3	A	26	2931.5721	1466.2897
327.2391	355.2340	178.1206	4	G	25	2860.5349	1430.7711
440.3231	468.3180	234.6627	5	L	24	2803.5135	1402.2604
577.3820	605.3770	303.1921	6	H	23	2690.4294	1345.7183
692.4090	720.4039	360.7056	7	D	22	2553.3705	1277.1889
793.4567	821.4516	411.2294	8	T	21	2438.3436	1219.6754
921.5152	949.5102	475.2587	9	Q	20	2337.2959	1169.1516
1034.5993	1062.5942	531.8007	10	I	19	2209.2373	1105.1223
1163.6419	1191.6368	596.3220	11	E	18	2096.1532	1048.5803
1220.6634	1248.6583	624.8328	12	G	17	1967.1107	984.0590
1462.8013	1490.7962	745.9017	13	K	16	1910.0892	955.5482
1575.8853	1603.8802	802.4438	14	L	15	1667.9513	834.4793
1712.9442	1740.9392	870.9732	15	H	14	1554.8672	777.9373
1859.9796	1887.9746	944.4909	16	M	13	1417.8083	709.4078
1931.0168	1959.0117	980.0095	17	A	12	1270.7729	635.8901
2032.0644	2060.0593	1030.5333	18	T	11	1199.7358	600.3715
2103.1015	2131.0965	1066.0519	19	A	10	1098.6881	549.8477
2216.1856	2244.1805	1122.5939	20	L	9	1027.6510	514.3291
2344.2442	2372.2391	1186.6232	21	Q	8	914.5669	457.7871
2457.3283	2485.3232	1243.1652	22	I	7	786.5084	393.7578
2528.3654	2556.3603	1278.6838	23	A	6	673.4243	337.2158
2656.4239	2684.4189	1342.7131	24	Q	5	602.3872	301.6972
2769.5080	2797.5029	1399.2551	25	L	4	474.3286	237.6679
2870.5557	2898.5506	1449.7789	26	T	3	361.2445	181.1259
2983.6397	3011.6347	1506.3210	27	L	2	260.1969	130.6021
			28	K	1	147.1128	74.0600

[Click] to move table

Modifications:
K: 114.042937 [13]
M: 15.994915 [16]

Supplementary Figure 6. MS/MS analysis of Rpn10 peptides. (a) The MS/MS spectrum of Rpn10 peptides containing K84 with a di-glycine (KGG) tag. **(b)** The MS/MS spectrum of Rpn10 peptides containing K84 with di-glycine (KGG) tag and Oxy-Met. The precursor peptide ions were isolated and fragmented in an LTQ mass spectrometer. Fragmentation ions from both termini are shown (b- and y-ions, respectively), where the detected fragmentation ions are colored in red (y-ions) and blue (b-ions).

His$_6$-Ub-GST-Rpn10 WT

Ubiquitylation site	Peptide count
K71	1
K84	14
K99	1
K268	5
Total	21

His$_6$-Ub-GST-Rpn10 mutant

ubiquitylation site	Peptide count
K268	3
Total	3

Ub-Rpn10 WT

ubiquitylation site	Peptide count
K71	1
K84	20
K99	0
K268	5
Total	26

Ub-Rpn10 mutant

ubiquitylation site	Peptide count
K268	2
Total	2

Supplementary Figure 7. List of detected peptides in MS experiments. The number and location of GG-peptides corresponding to the ubiquitylation sites on Rpn10 WT and mutant protein that were detected in MS experiments is shown.

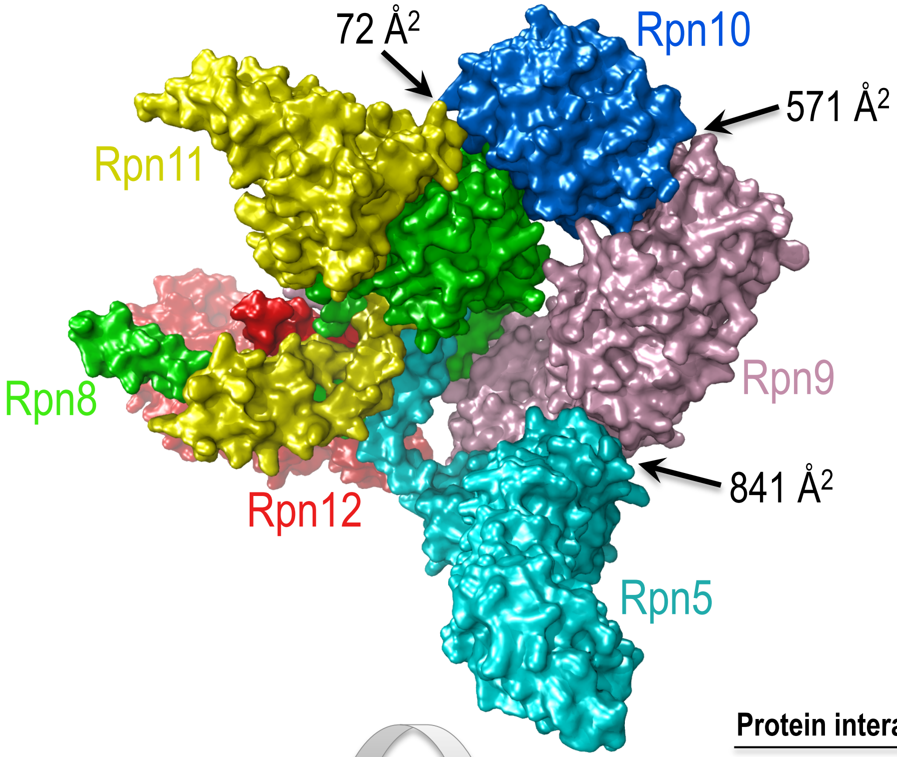

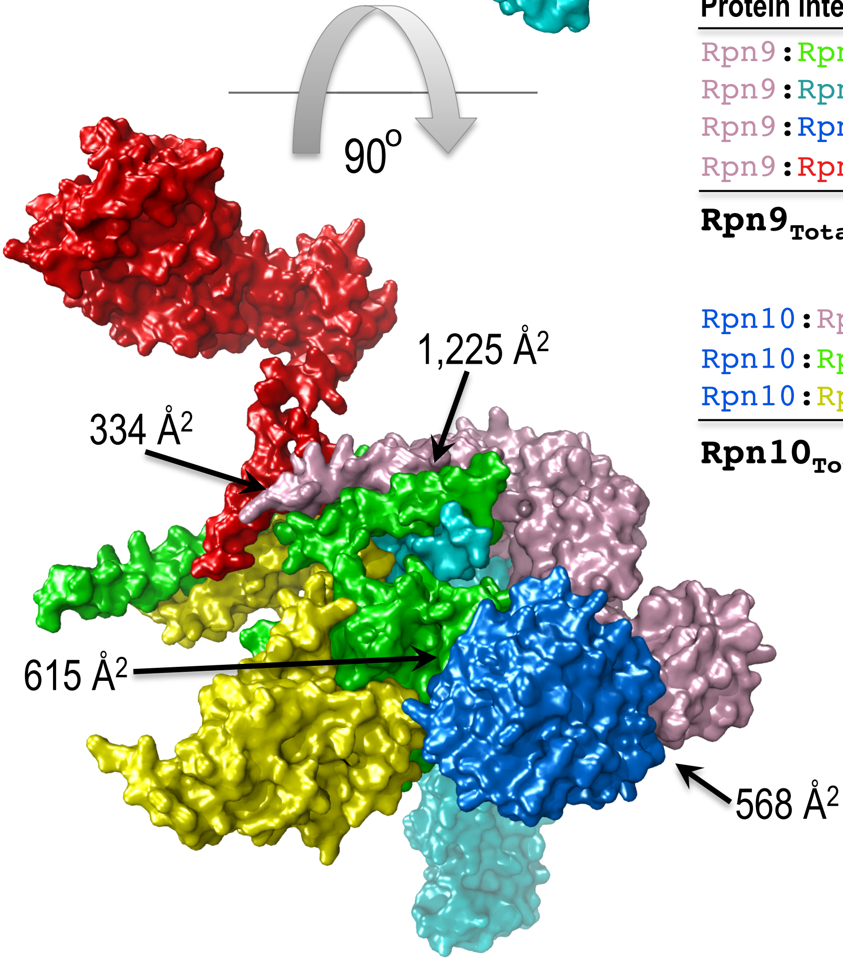

Protein interactions	BSA (Å2)
Rpn9:Rpn8	1,225
Rpn9:Rpn5	841
Rpn9:Rpn10	571
Rpn9:Rpn12	334
**$Rpn9_{Total}$ =**	**2,971**
Rpn10:Rpn9	568
Rpn10:Rpn8	615
Rpn10:Rpn11	72
**$Rpn10_{Total}$ =**	**1,225**

Supplementary Figure 8. Buried Surface Area (BSA) formed by the interactions of Rpn10 and Rpn9 with their neighbors. BSA were calculated using the PISA server at http://www.ebi.ac.uk/msd-srv/prot_int/cgi-bin/piserver
The coordinates of the proteasome EM structure 4CR2 were used as input for the PISA analysis. The surfaces of Rpn10, Rpn9 and their neighbors are presented with the indicated colors.

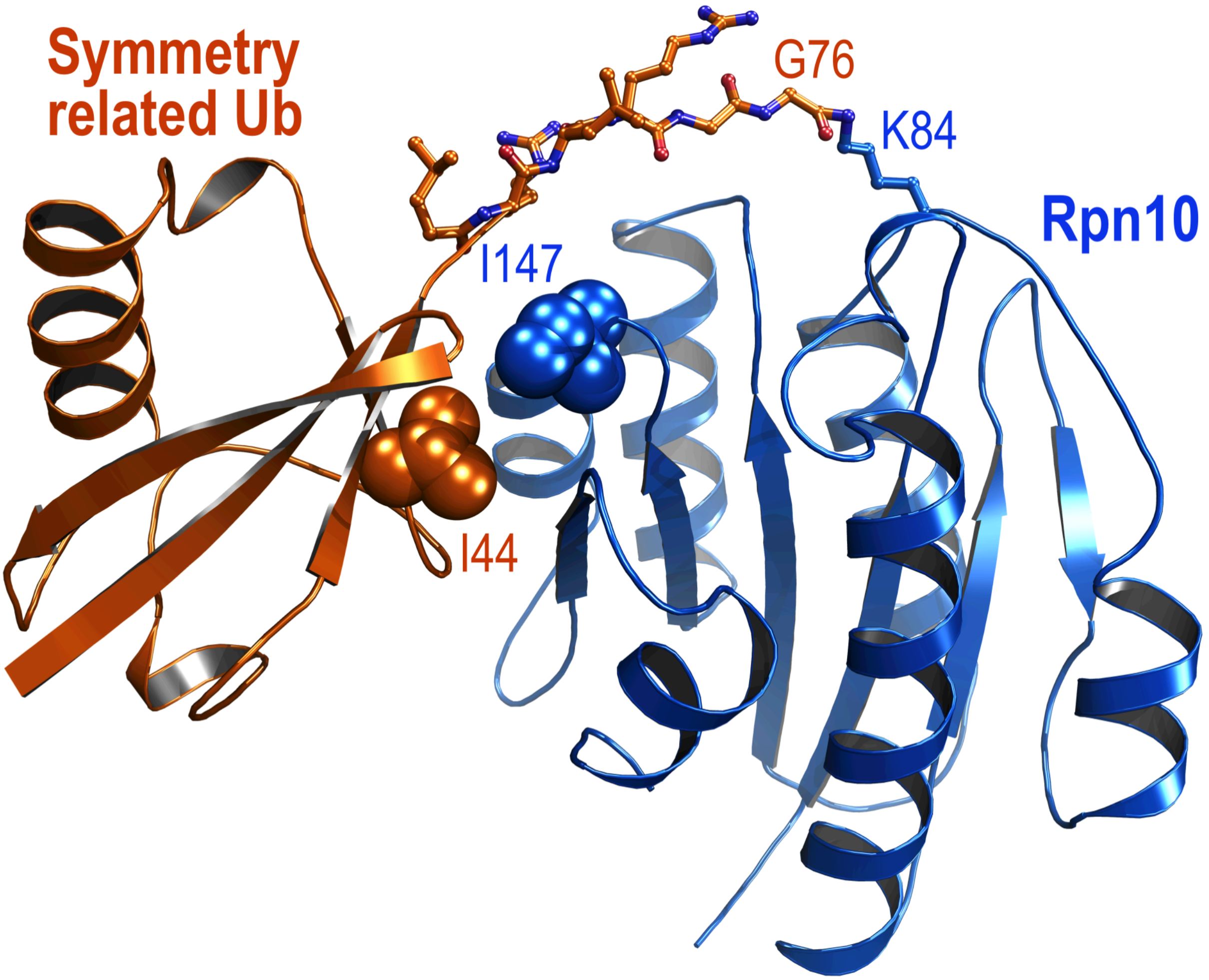

Supplementary Figure 9. The carboxyl terminus tail of the non-covalent bound Ub at the crystallographic symmetry can interacts with K84 to form an isopeptide bond

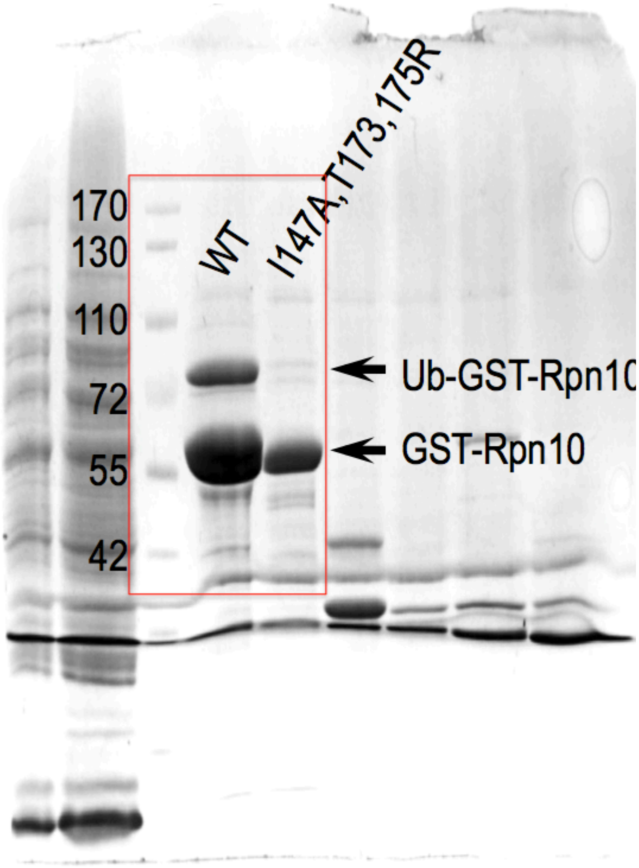

Fig. 3g

Supplementary Figure 10. Uncropped image of representative original Coomassie-blue stained SDS-PAGE. Red boxes indicate the cropped area displayed in the indicated figure.

Supplementary table 1: List of plasmids used in this study

**Name** [a]	**Ori** [b]	**Resistance**	**Promoter**	**Construct**	**Source**
pCOG6	ColE1	Amp [c]	Ptac	pGST-Rpn10-FL^{Sc}	Lab collection
pCOG30	ColE1	Amp	T7	pET43.1-NusA-Rsp5$_{(109\text{-}809)}^{Sc}$	Lab collection
pCOG31	CloDF13	Sm [d]	T7	pCDF-MBP-Rpn10^{Sc}	Lab collection
pCOG33	CloDF13	Sm	T7	pCDF-His$_6$-MBP-Rsp5^{Sc}	Lab collection
pCOG34	ColE1	Amp	Ptac	pGST-par2-VWA$_{1\text{-}189}^{Sc}$	This work
pCOG35	ColE1	Amp	Ptac	pGST-par2-VWA$_{1\text{-}189,\ T173R,T175R}^{Sc}$	This work
pCOG36	ColE1	Amp	Ptac	pGST-par2-VWA$_{1\text{-}189,\ I147A}^{Sc}$	This work
pCOG37	ColE1	Amp	Ptac	pGST-par2-VWA$_{1\text{-}189,\ Q149A}^{Sc}$	This work
pCOG38	ColE1	Amp	Ptac	pGST-par2-VWA$_{1\text{-}189,\ Q149R}^{Sc}$	This work
pCOG39	ColE1	Amp	Ptac	pGST-par2-Rpn10$_{190\text{-}268}^{Sc}$	This work
pCOG63	ColE1	Amp	Ptac	pGST-Rpn10-FL$_{I147A,T175R,T173R}^{Sc}$	This work
pCOG70	ColE1	Kan [e]	T7	pHis$_6$-par2-Rpn9^{Sc}	This work
pCOG71	ColE1	Kan	T7	pHis$_6$-par2-Rpn12^{Sc}	This work
pCOG72	ColE1	Kan	T7	pHis$_6$-Dsk2-UbL^{Sc}	Glickman lab
pGEN4	ColE1	Kan	T7	pHis$_6$-par2-Ub^{Hs}/Ubc4^{Sc}/ Uba1^{Ta}	Lab collection
pGEN24	ColE1	Tet [f]	T7	pHis$_6$-par2-Ub^{Hs}/Ubc4^{Sc}/ Uba1^{Ta}	Lab collection
pGEN25	ColE1	Kan	T7	pHis$_6$-par2-Ub^{Hs}	Lab collection
pGEN33	ColE1	Amp	T7	pHis$_6$-par1-Ub$_{L8E,I44E,V70D}^{Hs}$	Lab collection
pGEN35	ColE1	Kan	T7	par2-Ub^{Hs}	Lab collection
pCD-Sub13	CloDF13	Sm	pLtetO1	pCDF- cDHFR-link2-His$_6$-Rpn10^{Sc}	This work
pCD-Sub19	CloDF13	Sm	pLtetO1	pCDF- cDHFR-link2-His$_6$-MBP-Rpn10$_{1\text{-}189}^{Sc}$	This work
pCD-Sub40	CloDF13	Sm	pLtetO1	pCDF- cDHFR-link1-His$_6$-Rpn10$_{190\text{-}268}^{Sc}$	This work
pCD-Sub72	CloDF13	Sm	pLtetO1	pCDF- cDHFR-link2-His$_6$-MBP-Rpn10$_{1\text{-}189,T173R\ T175R}^{Sc}$	This work
pCD-Sub73	CloDF13	Sm	pLtetO1	pCDF- cDHFR-link2-His$_6$-MBP-Rpn10$_{1\text{-}189,I147R}^{Sc}$	This work
pCD-Sub74	CloDF13	Sm	pLtetO1	pCDF- cDHFR-link2-His$_6$-MBP-Rpn10$_{1\text{-}189,Q149R}^{Sc}$	This work

Name[a]	Ori[b]	Resistance	Promoter	Construct	Source
pCD-Sub75	CloDF13	Sm	pLtetO1	pCDF- cDHFR-link2-His_6-MBP-$Rpn10_{1-189,Q149A}^{Sc}$	This work
pND-Ub04	ColE1	Kan	pLtetO1	pZE21-nDHFR-link2-His_6-Ub^{Hs}/$Ubc4^{Sc}$/$Uba1^{Ta}$	This work
pND-Ub18	ColE1	Kan	pLtetO1	pZE21-nDHFR-link2-His_6-Ub^{Hs}	This work
pND-Ub22	ColE1	Kan	pLtetO1	pZE21-nDHFR-link2-$His_6Ub_{I44E,L8E,V70D}$/$UBC4^{Sc}$/$Uba1^{Ta}$	This work
pND-Ub44	ColE1	Kan	pLtetO1	pZE21-nDHFR-link2-His_6-Ub_{I44E}/$UBC4^{Sc}$/$Uba1^{Ta}$	This work
pND-Ub55	ColE1	Kan	pLtetO1	pZE21-nDHFR-link2-His_6-Ub_{H68L}/$UBC4^{Sc}$/$Uba1^{Ta}$	This work
pGST-DHFR	ColE1	Amp	Ptac	pGST-$DHFR^{Mm}$	This work
pCOG3	ColE1	Amp	Ptac	pMBP-par1-$RSP5^{Sc}$	Lab collection

[a] Our laboratory collection identifier; [b] Origin of replication; [c] Amp, ampicillin; [d] Sm, streptomycin; [e] Kan, kanamycin, [f] Tet, tetracycline, *Hs-Homo sapiens, Mm-Mus musculus, Sc- Saccharomyces cerevisiae, Ta-Triticum aestivum*.
